# *Sarcopoterium spinosum* extract improved insulin sensitivity in mice models of glucose intolerance and diabetes

**DOI:** 10.1371/journal.pone.0196736

**Published:** 2018-05-16

**Authors:** Konstantin Rozenberg, Tovit Rosenzweig

**Affiliations:** Departments of Molecular Biology and Nutritional Studies, Ariel University, Ariel, Israel; Consiglio Nazionale delle Ricerche, ITALY

## Abstract

**Background:**

The glucose lowering properties of *Sarcopoterium spinosum*, a traditional medicinal plant, were previously validated by us using KK-Ay mice as a genetic model for type 2 diabetes (T2D).

**Objective:**

To clarify the effects of *Sarcopoterium spinosum* extract (SSE) on diet-induced glucose intolerance and to investigate SSE effects on carbohydrate and lipid metabolism in target tissues of both high-fat-diet (HFD)-fed and KK-Ay mice.

**Results:**

Mice were given SSE (70 mg/day) for 6 weeks. SSE improved glucose tolerance and insulin sensitivity in HFD-fed mice as was demonstrated previously in KK-Ay mice. Higher insulin sensitivity was validated by lower serum insulin and activation of the insulin signaling cascade in skeletal muscle and liver of SSE-treated mice in both models. H&E staining of the livers demonstrated lower severity of steatosis in SSE-treated mice. Several model-specific effects of SSE were observed–mRNA expression of proinflammatory genes and CD36 was reduced in SSE-treated KK-Ay mice. Hepatic mRNA expression of PEPCK was also reduced in SSE-treated KK-Ay mice, while other genes involved in carbohydrates and lipid metabolism were not affected. HFD-fed mice treated by SSE had elevated hepatic glycogen stores. Gluconeogenic gene expression was not affected, while GCK expression was increased. HFD-induced hepatic steatosis was not affected by SSE. However, while genes involved in lipid metabolism were downregulated by HFD, this was not found in HFD-fed mice given SSE, demonstrating an expression profile which is similar to that of standard diet-fed mice.

**Conclusion:**

Our study supports the insulin sensitizing activity of SSE and suggests that this extract might improve other manifestations of the metabolic syndrome.

## Introduction

Diabetes mellitus (DM) has reached epidemic proportions, affecting more than 170 million individuals worldwide and the number of affected subjects is expected to further increase in the coming years [1]. The most common form of DM is type 2 diabetes (T2D), accounting for more than 90% of diabetes cases. T2D is considered to be one of the major causes for premature illness and death in the western world [2,3]. The increased morbidity and mortality associated with T2DM is a result of chronic hyperglycemia, dyslipidemia, low-grade chronic inflammation, and other metabolic disturbances accompanying the disease.

Several drugs, acting via various mechanisms, are approved for treatment of diabetes. However, despite the availability of these medications acting on different targets, around 50% of patients failed to maintain glycemic control according to the treatment's goals as recommended by international diabetic associations [4,5]. These data further illustrate the continual need for additional research and development of alternative drugs with novel mechanisms to slow disease progression and its related complications.

In recent years, considerable attention has been directed to identify plants with anti-diabetic activity. Such plants may be consumed as medicinal herbs to improve glycemic control or its active constituents isolated and identified in order to develop a chemical drug. Even with the presence of ethno-pharmacological evidence for the use of certain herbs for medicinal purposes, most regulatory agencies demand both preclinical and clinical efficacy data to approve herbs for human consumption [6]. Accordingly, there is a need for intense, thorough, and wide research on candidate plants with putative antidiabetic activity. Approximately 1000 plants are reported to have been used to treat diabetes, based on ethnobotanical information [7]. Experimental validation of anti-diabetic action has been performed on around 400 medicinal plants. However, extensive research, including *in-vivo* experiments, using appropriate models of T2D and exploration of the mechanism of action, is available for the minority of these herbs [8–10]. Moreover, results of well-designed clinical trials are available only for a very limited number of these traditionally used medicinal herbs, with major variability in results. As a result, the number of anti-diabetic plants with a strong scientific basis supporting their safety and efficacy is extremely limited.

The antidiabetic activity of *Sarcopoterium spinosum* root extract (SSE) was reported by several ethnobotanical surveys [11–14] and was already demonstrated by us using *in-vitro* models [15,16]. Antidiabetic properties were also found in extracts of aerial parts of *S*. *spinosum* [17]; however, root extract, which is the organ recommended for use by traditional medicine, exerts the most potent effects. In addition, improved glucose tolerance and insulin sensitivity were found in a genetic mice model of type 2 diabetes treated by this extract [15,16]. As T2D is a disease developed in genetic-susceptible subjects as a result of exposure to a diabetogenic environment, it is important to clarify the potential effects of SSE on diet-induced glucose intolerance. In this study, the effect of SSE on glucose tolerance in diet-induced insulin-resistant mice is investigated for the first time. In addition, the effect of SSE on metabolic pathways involved in carbohydrates and lipids metabolism in skeletal muscle and liver of two mice models of insulin resistance, both genetic and diet-induced, is analyzed.

## Materials and methods

### Chemicals reagents and antibodies

D-Glucose and insulin were purchased from Sigma. Protease inhibitors were purchased from Merck. The following antibodies was purchased from Cell Signaling Technologies: Monoclonal rabbit anti-phospho-insulin receptor β (Tyr1150/1151, #3024), monoclonal rabbit anti-insulin receptor β (#3025, dilution used 1:1000), polyclonal rabbit anti-phospho-PKB (Ser473, #9271), monoclonal rabbit anti-PKB (pan, #4685), monoclonal rabbit anti-phospho-GSK3β (Ser9) (#5538), monoclonal mouse anti-GSK3β (#9832), and monoclonal mouse anti-phospho-PRAS40 (Thr246 (#13175S), all diluted at 1:1000. A monoclonal mouse anti-Actin was obtained from MP Biomedicals (#69100) and used in 1:10000 dilution. Secondary antibodies were purchased from Jackson Immuno Research, Peroxidase conjugated affiniPure goat anti mouse (#115-035-003) and Peroxidase conjugated affiniPure goat anti rabbit (#111-035-003) both were used in 1:1000 dilution.

### Methods

#### *S*. *spinosum* extract preparation

*Sarcopoterium spinosum* (L.) Spach. [Thorny burnet, local name: Natesh, Billan (Arabic), Sira Kotzanit (Hebrew), the plant name has been checked with http://www.theplantlist.org.] was collected from the wild in the area around Ariel University, in accord with the laws of Israel' authorities for biodiversity. The plants were identified by Dr. Dvir Taler, an agronomics and botanist consultor, based on morphological and binocular analyses. A voucher specimen of the plant was deposited in the Israel National Herbarium at the Hebrew University of Jerusalem (No. HUJ 102531). *S*. *spinosum* aqueous root extract was prepared, as described previously [15,16], by boiling 100 g roots/L. The extract was lyophilized and kept at -20°C, giving a yield of 0.7% dry material. To ensure the uniformity of the extract, the herbs used for the study were of the same ecotype throughout the study and were uprooted during the same season. The uniformity of the extracts was maintained by performing a set of measurements, including several bioassays that were developed based on the biological activity of the extract and RPLC analysis, as described previously [17]. Catechin level was quantified based on calibration curve, and found to be at a concentration of 7μg/mg dry extract.

#### Experimental protocol

The protocol of the study was approved by the Committee on the Ethics of Animal Experiments of the University of Ariel (Permit Number: IL-73-09-15). The Animal House in Ariel University operates in compliance with the rules and guidelines of the Israel Council for Research on Animals, based on the US NIH Guide for the Care and Use of Laboratory Animals. The mice were housed in an animal laboratory with a controlled environment of 20–24°C, 45–65% humidity, and a 12 h light/dark cycle. Animals had been anesthetized by ketamine + xylazine as required, and all efforts were made to minimize suffering.

The study was performed on KK-Ay mice, a genetic model of T2D and on a model of diet-induced glucose intolerance, using high fat diet-fed C57bl/6 mice (HFD, 60% of total calories derived from fatty acids, 18.4% from proteins, and 21.3% from carbohydrates, Envigo, Teklad TD.06414). For these experiments, 6 week old male mice were separated into treatment groups, 8–10 mice each. KK-Ay male mice were separated into 2 groups as follows: control-untreated mice and SSE-treated mice. The mice were fed with standard (STD) diet (18% of total calories derived from fat, 24% from proteins, and 58% from carbohydrates. Harlan, Teklad TD.2018). C57Bl/6J mice were purchased from Envigo (Israel). C57bl/6 male mice were separated into 3 treatment groups, 8–10 mice each, as follows: control mice fed with STD or HFD, and HFD-fed mice supplemented with *S*. *spinosum* extract. SSE (35 mg/day or 100 mg/day dry material in C57BL6/J and KK-Ay mice, respectively) was administered daily in the drinking water starting at age 6 weeks. Body weight was measured once a week. At age of 12 or 17 weeks in the KK-Ay or C57Bl/6J, respectively, mice were anesthetized using ketamine + xylazine and euthanized by terminal bleeding followed by cervical dislocation. Blood was collected from the heart and serum was prepared. Serum insulin was measured by immunoassay, using a commercial ELISA kit (Mercodia, Sweden). Livers were perfused and both liver and soleus muscle were isolated. In order to follow insulin-induced protein phosphorylation in liver and skeletal muscle, in some of the mice (n = 5), insulin was injected (0.75 mU /g body weight) 15 min before killing the animal. Liver and muscle were snap frozen in liquid nitrogen, and preserved in -80°C for later protein and RNA extraction. Part of the livers were saved in 4% paraformaldehyde for histological analyses.

#### Glucose tolerance test (GTT)

Intraperitoneal glucose tolerance test (GTT) was performed on C57bl/6 mice at age 15 weeks. Mice were injected with 1.5 mg glucose/g body weight after 6-h fast. Blood glucose was determined from tail blood using the ACCU-CHEK Go glucometer (Roche, Germany).

#### Western blot analysis

Protein lysates were prepared using RIPA buffer supplemented with protease and phosphatase inhibitors. The samples were homogenized and centrifuged at 14,000 rpm for 20 min. The supernatant was collected and protein concentration was measured using the Bradford method. 20 μg protein per lane was separated by SDS-polyacrylamide gel electrophoresis. Proteins were electrophoretically transferred onto nitrocellulose membranes. The membranes were blocked in 5% dry milk, incubated with the appropriate antibodies solutions (5% BSA in 0.01% TBST) and proteins were immunodetected using the enhanced chemiluminescence method.

#### Analysis of mRNA expression by real-time PCR

Total RNA was extracted from the liver using TRI reagent (Molecular Research Center, Inc. Cincinnati, OH) according to manufacturer instructions. Of total RNA, 3 μg were reverse transcribed by oligo-dT priming (Stratascript 5.0 multi-temperature reverse transcriptase, Stratagene) according to manufacturer instructions. Real-time PCR amplification reactions were performed using SYBR Fast Universal Ready-mix Kit (Kappa Biosystems), by the MxPro QPCR instrument (Stratagene). Primers for real time PCR reactions were synthesized by Sigma, Israel. Primer sequences are listed in Table 1.

**Table 1:** Primers list.

Gene	Forward	Reverse
PPARα	ATGCCAGTACTGCCGTTTTC	CCGAATCTTTCAGGTCGTGT
PPARγ	CAGGCCTCATGAAGAACCTT	ACCCTTGCATCCTTCACAAG
SREBP2	AGAGGCGGACAACACACAAT	ACGCCAGACTTGTGCATCTT
SREBP1c	AAGAGCCCTGCACTTCTTGA	CCACAAAGAAACGGTGACCT
HSL	TGCTCTTCTTCGAGGGTGAT	TCTCGTTGCGTTTGTAGTGC
ACC1	CATGAACACCCAGAGCATTG	ATTTGTCGTAGTGGCCGTTC
FAS	TTGCTGGCACTACAGAATGC	AACAGCCTCAGAGCGACAAT
AdipoR1	TCGTGTATAAGGTCTGGGAG	GCAGATGTGTCCAGATGTTG
AdipoR2	CCCAGGAAGATGAAGGGTTT	TTAAGCCAATCCGGTAGCAC
CD36	AGCAGCTGCACCACATATCTAC	GGAACCAAACTGAGGAATGG
GCK	GCAGAAGGGAACAACATCGT	TGGCGGTCTTCATAGTAGCA
PEPCK	AGCCTTTGGTCAACAACTGG	GTTATGCCCAGGATCAGCAT
G6Pase	GATTCCGGTGTTTGAACGTC	GTAGAATCCAAGCGCGAAAC
GLUT-2	TGGGCTAATTTCAGGACTGG	TGCCCAGAATAAAGCTGAGG
CPT-1	GATGTGGACCTGCATTCCTT	TCCTTGTAATGTGCGAGCTG
HMGCR	CGTAACCCAAAGGGTCAAGA	GACAGCCAAAAGGAAGGCTA
AdipoR1	TCGTGTATAAGGTCTGGGAG	GCAGATGTGTCCAGATGTTG
AdipoR2	CCCAGGAAGATGAAGGGTTT	TTAAGCCAATCCGGTAGCAC
PAI-1	GGGCAAACAGTTGCGTAAAG	TGTTGATCGGGTCTTCTTCC
MCP-1	CACTCACCTGCTGCTACTCATT	TCTGGACCCATTCCTTCTTG
CRP	AGATCCCAGCAGCATCCATA	TCTGCTTCCAGAGACACATAGG
IKK	CACGTTGGACATGGATCTTG	TTCCTCAGCTGGAAGAAGGA
HPRT	TGTTGTTGGATATGCCCTTG	TTGCGCTCATCTTAGGCTTT

#### Hepatic triglyceride (TG) content

One hundred milligrams of liver were homogenized in 1 ml water solution containing 5% NP-40. The samples were twice heated to 80–100°C for 5 min and cooled to room temperature. The sample was centrifuged for 2 min and the supernatant was used for TG analysis using a Triglyceride quantification kit (Abcam, Cambridge, UK) according to manufacturer instructions.

#### Hepatic total cholesterol levels

Ten milligrams of liver were homogenized in a solution of Chloroform: Isopropanol: NP-40 (7:11:0.1). The organic phase was collected and vacuum dried for about 2 h, resuspended in a cholesterol assay buffer supplied by Abcam and used in a Cholesterol/Cholesteryl Ester quantitation kit (Abcam, Cambridge, UK) according to manufacturer instructions.

#### Hepatic glycogen content

Ten milligrams of liver were homogenized in a glycogen hydrolysis buffer supplied by Abcam. The sample was centrifuged for 5 min and the supernatant was used for glycogen analysis using the Glycogen Assay Kit II (Abcam, Cambridge, UK) according to manufacturer instructions.

#### Histochemistry

Livers were perfused, isolated, fixed in 4% paraformaldehyde and embedded in paraffin. Consecutive 4 μm sections were cut and stained with hematoxylin and eosin (H&E). A steatosis score was attained through blinded evaluation by a pathologist. Scoring of liver sections was carried out according to Modified Brunt criteria of staging and grading of non-alcoholic fatty liver disease (NAFLD) [18].

#### Statistical analysis

Values are presented as mean± SEM. Statistical differences between the treatments and controls were tested by unpaired two-tailed Student's t-test or one-way analysis of variance (ANOVA), followed by Bonferroni's post-hoc testing when appropriate. Analysis was performed using the GraphPad Prism 5.0 software. A difference of p<0.05 or less in the mean values was considered statistically significant.

## Results

### *Sarcopoterium spinosum* improved glucose tolerance in HFD-fed mice

**Fig 1:**
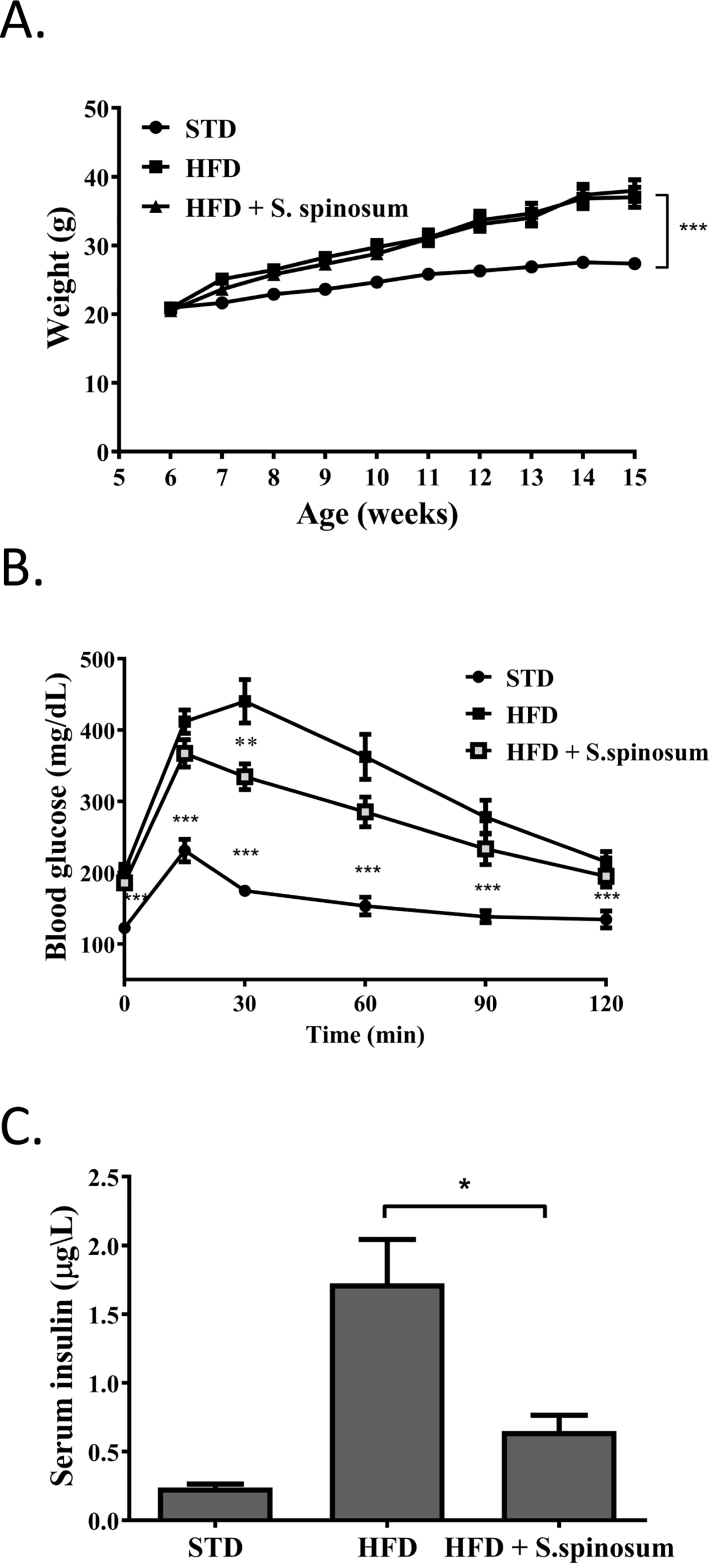
*S*. *spinosum* improved glucose tolerance in HFD-fed mice. C57BL/J mice were fed STD or HFD with or without SSE given in their drinking water as described in *Methods*. (A) Body weight was measured every week. (B) GTT was performed at age 15 weeks as described in *Materials and Methods*. (C) Fasting serum insulin levels were measured at age 17 weeks. The result are presented as mean±SE, *p<0.05, **p< 0.005, ***p< 0.0005 by Student's *t*-test, compared to HFD-fed mice.

HFD consumption is known to induce alteration in insulin sensitivity leading to glucose intolerance and a state of prediabetes [19]. HFD-fed mice were given SSE for 10 weeks and the effects on body weight and glucose homeostasis were followed. Food consumption and drinking habits were measured demonstrating lower food consumption in HFD-fed groups and no difference between all 3 groups in drinking habits (data not shown). Body weight was higher in HFD-fed mice, with no effect of *S*. *spinosum* on the rate of body weight accumulation (Fig 1A). Fasting blood glucose and glucose disposal rate following intraperitoneal glucose load was altered in HFD-fed mice. SSE did not affect fasting glucose levels but improved glucose tolerance of the HFD-fed mice (Fig 1B), an effect that was accompanied by reduced fasting serum insulin levels (Fig 1C). These results indicate a positive effect of SSE on the pre-diabetic hyper-insulinemic HFD- fed mice.

### *S*. *spinosum* improved insulin signaling in soleus muscle and liver of HFD- fed mice

**Fig 2:**
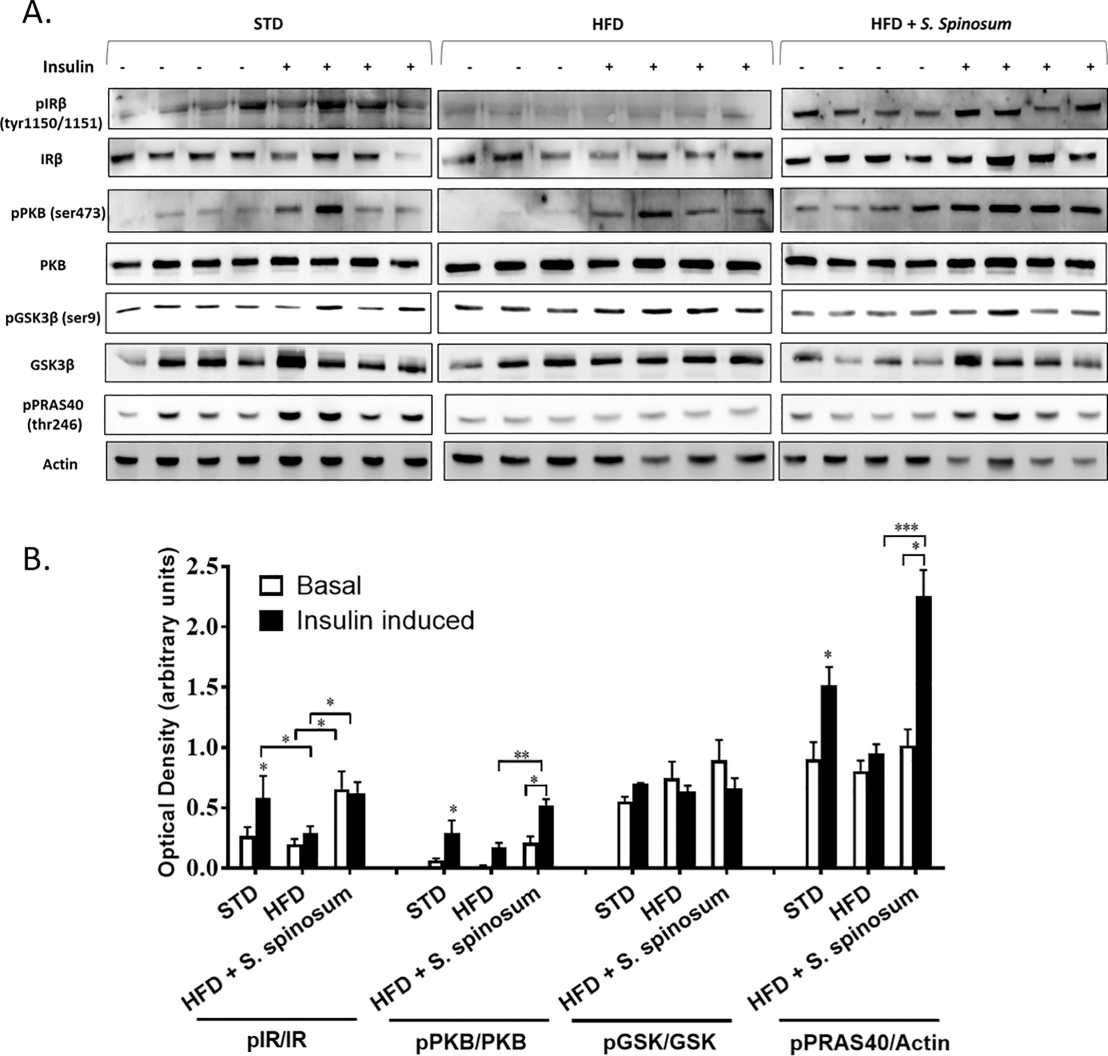
*S*. *spinosum* enhances insulin signaling in muscle of HFD-fed mice. C57BL/J mice model were fed STD, HFD or HFD+ *S*. *spinosum*. Soleus muscle was removed at the age of 17 weeks as described in *Methods*. (A) Western blot analysis was performed on protein lysates of muscle using specific antibodies. (B) The bar graphs are the results of optical density measurements of western blots. Each bar represents the mean±SE of data obtained from 4 mice. *p<0.05 **p< 0.005 and ***p< 0.0005 compared to basal state in STD-fed mice or as indicated in graph, in One-way Anova followed by Bonferroni's post-test.

**Fig 3:**
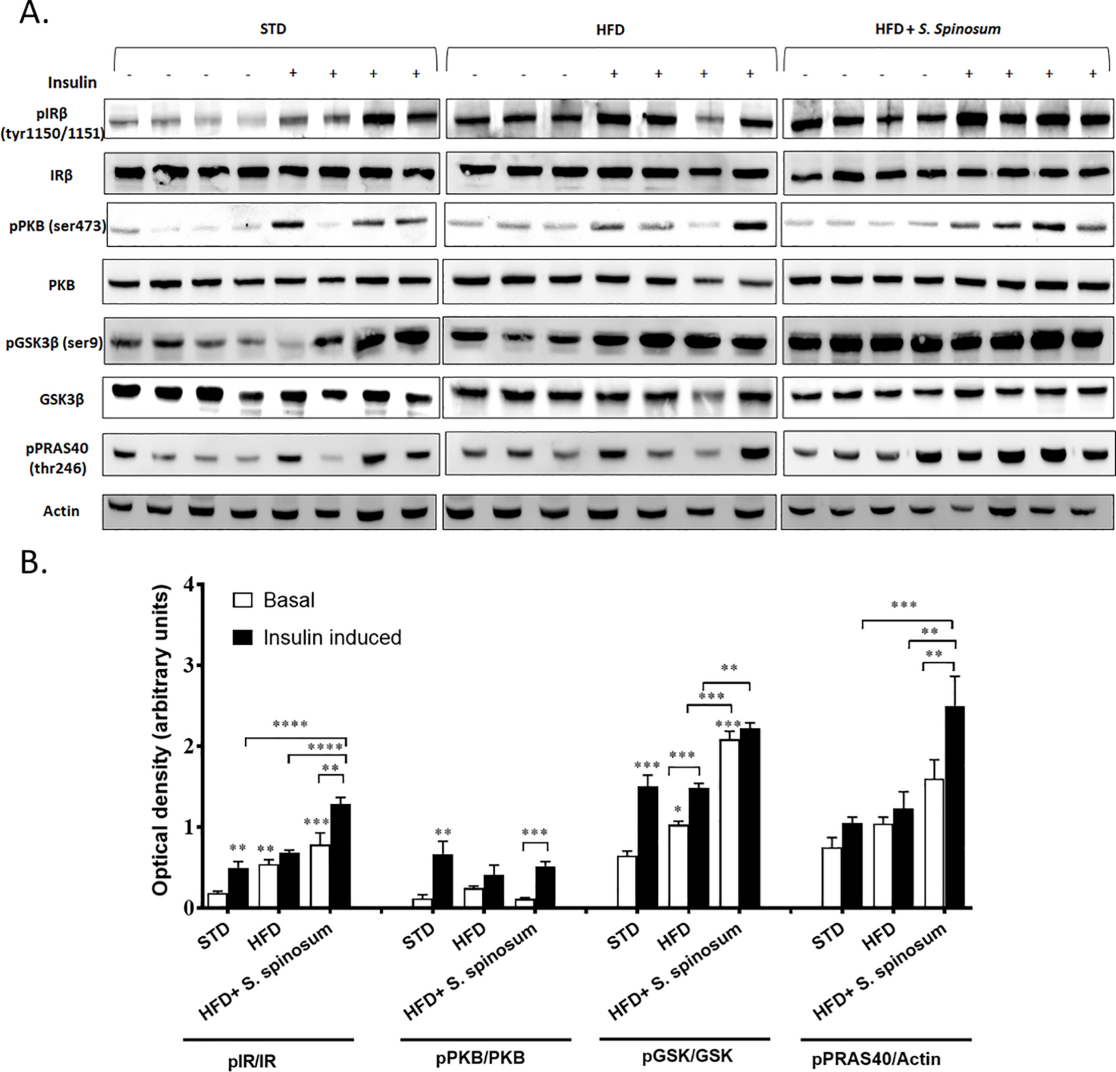
*S*. *spinosum* enhances insulin signaling in liver of HFD-fed mice. C57BL/J mice model were fed STD, HFD or HFD+ *S*. *spinosum*. Liver was removed at the age of 17 weeks as described in *Methods*. (A) Western blot analysis was performed on protein lysates of liver using specific antibodies. (B) The bar graphs are the results of optical density measurements of western blots. Each bar represents the mean±SE of data obtained from 4 mice. *p<0.05, **p< 0.005 and ***p< 0.0005 compared to basal state in STD-fed mice or as indicated in graph, in One-way Anova followed by Bonferroni's post-test.

In order to determine whether the improvement in glucose tolerance in HFD- fed mice supplemented by SSE is mediated by elevated activation of the insulin signaling cascade, the phosphorylation of key proteins mediating insulin signal transduction was followed in the soleus muscle and liver. HFD abolished insulin-induced phosphorylation of IR and PRAS40 in soleus muscle, while a residual phosphorylation of PKB remains (Fig 2). A significant increase in insulin-induced phosphorylation of IR, PKB and PRAS40 was demonstrated in SSE-treated mice compared to their HFD-fed littermates (Fig 2). Interestingly, basal IR phosphorylation was increased by SSE as well. On the other hand, HFD feeding increased basal phosphorylation of IR and GSK in the liver. SSE increased further basal phosphorylation of GSK3β (Fig 3), as well as insulin-induced phosphorylation of IR and PRAS40, while PKB was almost not affected either by HFD or by SSE. These findings revealed that SSE increased the activation of insulin signaling cascade in the muscle and the liver, in a tissue-specific manner.

### Effect of SSE on hepatic glycogen and lipid storage

**Fig 4:**
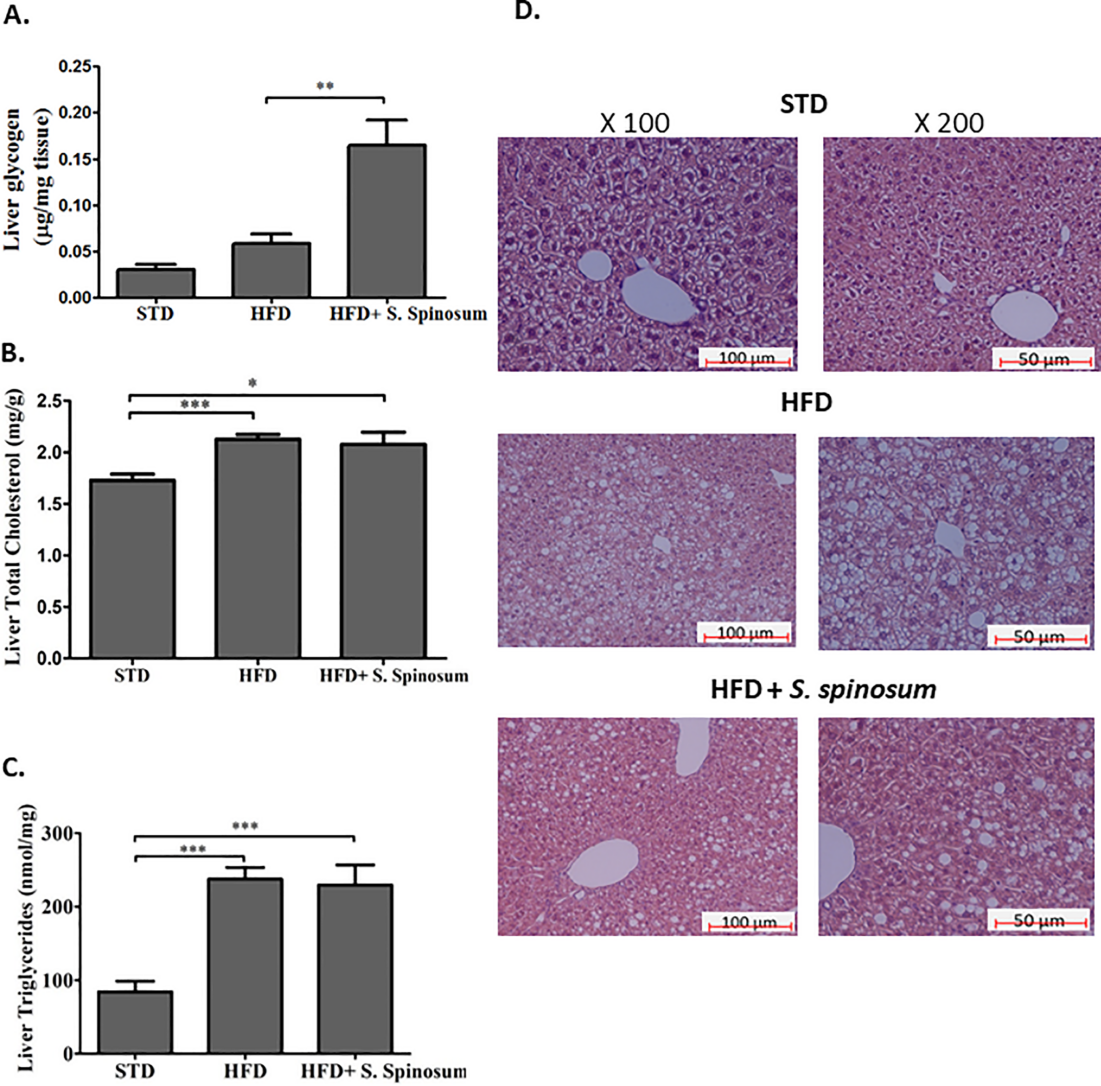
The effect of SSE on hepatic glycogen and lipid content in HFD-fed mice. C57BL/6J mice were fed STD, or HFD with or without SSE given in their drinking water as described in *Methods*. Mice were sacrificed at age 17 weeks and hepatic glycogen (A), triglycerides (B), and total cholesterol (C) levels were measured as described in *Materials and Methods*. The results are presented as mean±SE (n≥5 mice). *p<0.05, **p<0.005 by Student's *t*-test, compared to HFD-fed mice. (D) A representative H&E staining of livers.

Glycogen levels in liver were higher in HFD-fed mice supplemented by SSE (Fig 4A). This increase in carbohydrate stores is in accord with the high phosphorylation level of GSK3β demonstrated in these mice (Fig 3).

On the other hand, triglyceride and cholesterol levels were not affected by *S*. *spinosum* treatment; both control and *S*. *spinosum* groups of HFD-fed mice had elevated lipid levels in the liver (Fig 4B and 4C). However, H&E staining of the livers shows that steatosis is mainly localized to the periacinar area in *S*. *spinosum*-treated mice, compared to HFD-fed littermates, presenting steatosis extending into midzonal areas (Fig 4D).

### Effect of SSE on gene expression in livers of HFD-fed mice

**Fig 5:**
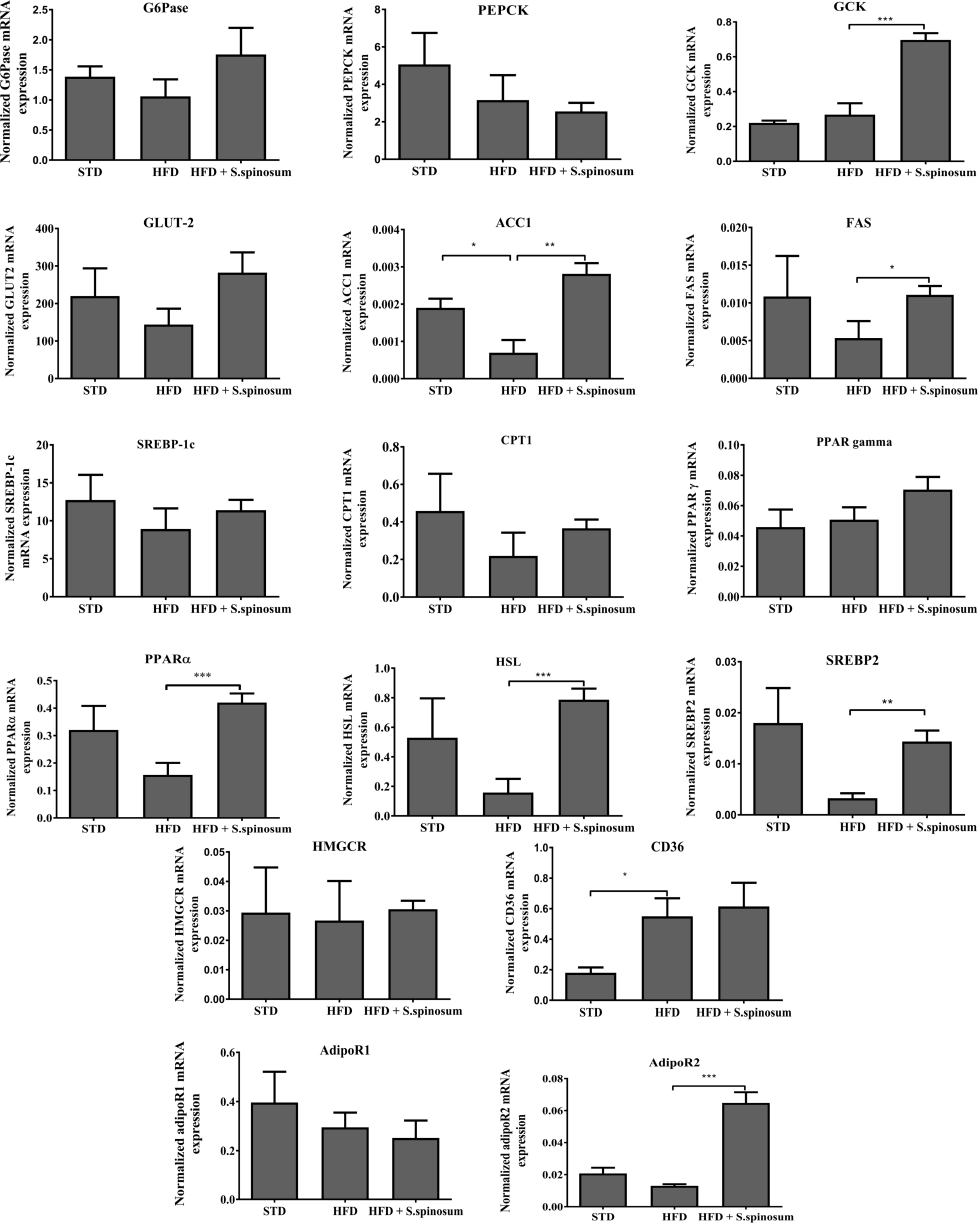
Effects of SSE on mRNA expression of hepatic genes. C57BL/J mice model were fed STD, HFD or HFD+ *S*. *spinosum*. Liver was removed at age 17 weeks as described in *Methods*. mRNA expression of selected genes was measured. Results were normalized to the expression of the housekeeping gene, HPRT. ***P<0.0005 by Student's *t*-test.

In order to support the results suggesting beneficial effects of the extract on liver, we measured the mRNA expression of several genes involved in glucose and lipid metabolism (Fig 5). While mRNA expression of gluconeogenic genes (*G6Pase* and *PEPCK*) was not affected either by diet or by *S*. *spinosum* treatment, the expression of *GCK*, encoding for the key enzyme of glycolysis, was increased in HFD-fed mice supplemented by SSE. *Glut2* mRNA expression was not significantly affected by the diet or the extract.

Regarding the expression of genes regulating lipid metabolism, while some genes were neither affected by the diet nor the extract (*PPAR*γ, *HMGCR*, SREBP-1C), several other genes were downregulated by HFD, including lipogenic genes (*ACC1*, *FAS* and *SREBP2*) as well as gene involved in lipolysis (*HSL*). The expression of *PPAR*α, a key master regulator in lipid metabolism and oxidation was also reduced in HFD-fed mice. The expression of all these genes was increased by *S*. *spinosum* to the same expression level presented in STD-fed mice.

*CD36*, which is associated with atherogenic processes and other features of the metabolic syndrome, was elevated in HFD-fed mice, with no effect of *S*. *spinosum* on its expression. On the other hand, mRNA expression of *AdipoR2*, the adiponectin receptor predominantly expressed in the liver, was increased in HFD-fed mice treated by *S*. *spinosum*. No difference was found in the expression of pro-inflammatory genes (*MCP-1*, *CRP*, *PAI-1* and *IKK*) between STD and HFD-fed groups (data not shown).

### Effects of SSE on target tissues of insulin in KK-Ay mice

The anti-diabetic properties of SSE were previously validated by us using the KK-Ay mice, a spontaneously (genetic) developing diabetes model. An improved glucose tolerance, accompanied by reduced serum insulin was found in *S*. *spinosum* treated mice [16], indicating that the extract affects the target tissues of insulin either by alleviating insulin resistance or by mimicking its action. In order to further support these findings, in the following experiments we investigated the effects of SSE on insulin signaling in target tissues of the hormone; the liver and skeletal muscle.

**Fig 6:**
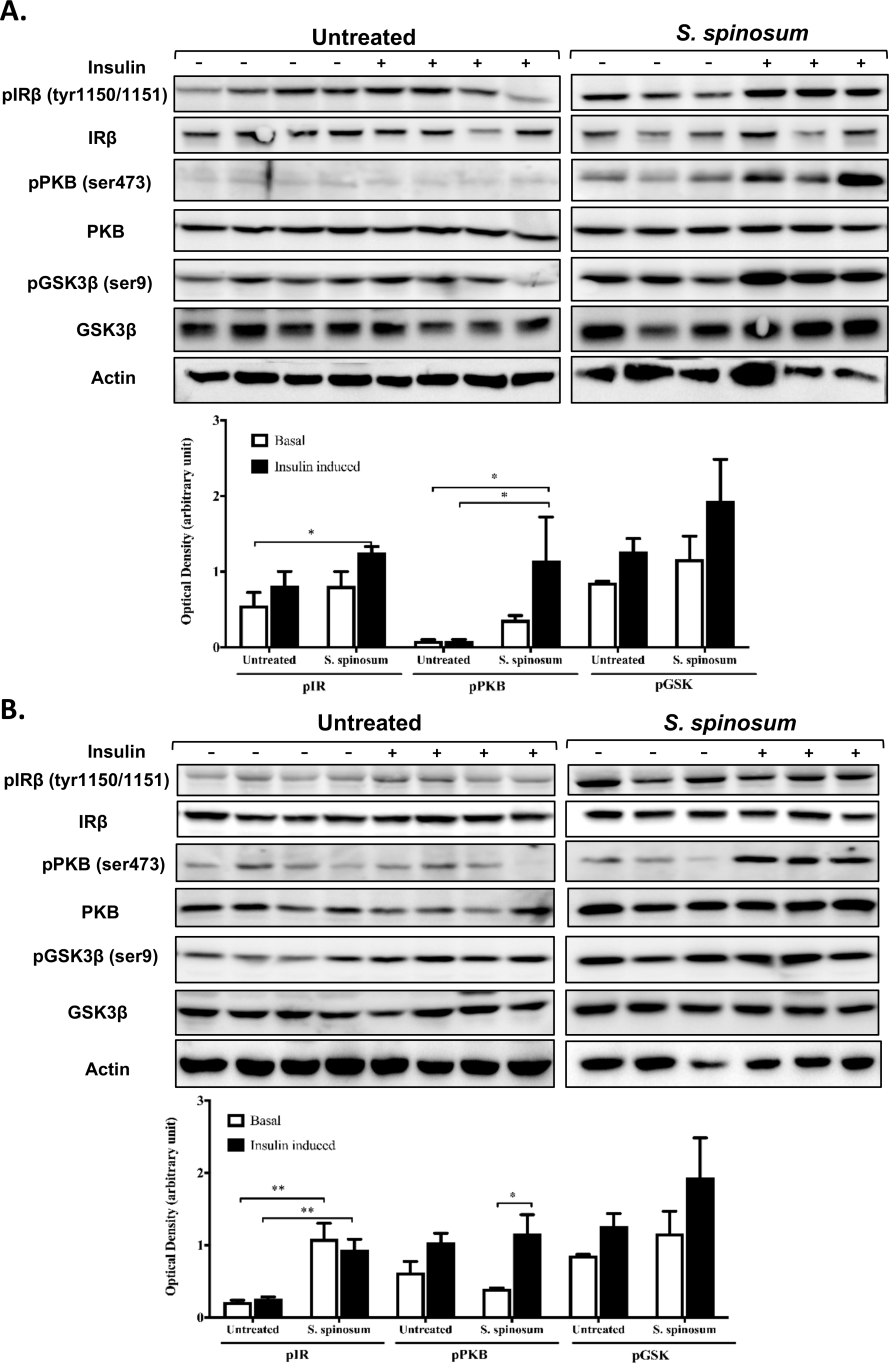
*S*. *spinosum* enhances insulin signaling in muscle and liver of KK-Ay mice. KK-Ay mice were given SSE for 6 weeks, soleus muscle and livers were removed at the age of 12 weeks as described in *methods*. Western blot analysis was performed on protein lysates of muscle (A) and liver (B) using specific antibodies. These are representative results of 3 independent experiments. The bar graph are the results of optical density measurements of western blots. Each bar represents the mean±SE of data obtained from 4 mice. *p<0.05 and **p< 0.005 in One-way Anova, followed by Bonferroni's post-test.

In the soleus muscle, insulin-induced phosphorylation of IR, PKB, and GSK3β was enhanced in *S*. *spinosum*-treated mice (Fig 6A). In the liver (Fig 6B), phosphorylation of the insulin receptor was elevated in *S*. *spinosum* treated mice in both basal and insulin-induced states. Insulin failed to induce PKB phosphorylation in control-untreated mice, while *S*. *spinosum* restores insulin-dependent PKB phosphorylation. GSK3β phosphorylation in the liver was not affected significantly by *S*. *spinosum* in this model.

### Hepatic glycogen and lipid accumulation

**Fig 7:**
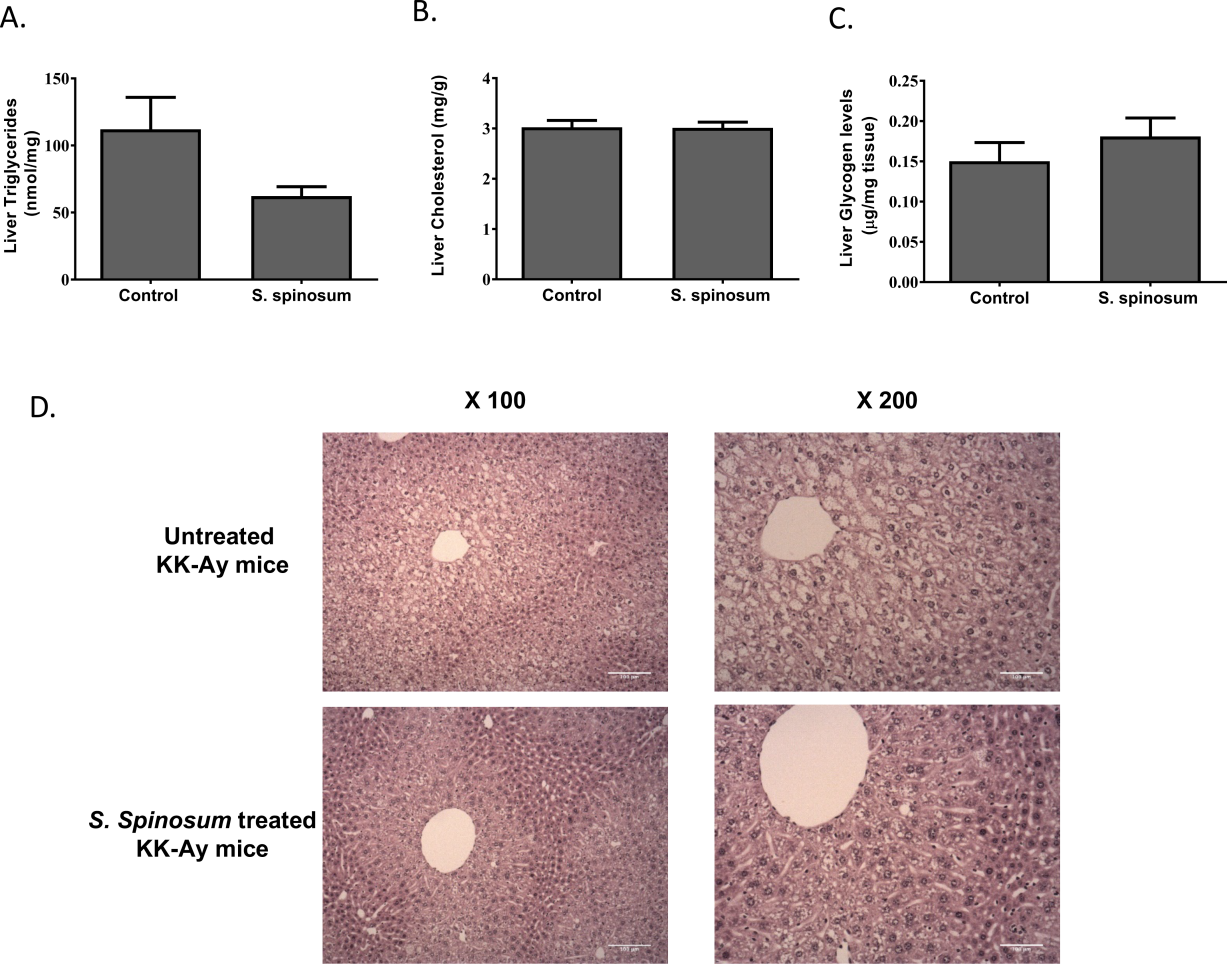
The effect of SSE on hepatic glycogen and lipid content in KK-Ay mice. KK-Ay mice were given SSE for 6 weeks as described in methods. Mice were sacrificed at age of 12 weeks and hepatic glycogen (A), triglycerides (B) and total cholesterol (C) levels were measured as described in *Materials and Methods*. The results are presented as mean±SE (n≥5 mice). *p<0.05, **p<0.005 by Student's *t*-test, compared to HFD-fed mice. (D) A representative H&E staining of livers.

Hepatic glycogen and triglycerides levels were measured (Fig 7). No difference was found in glycogen levels between groups, in accord with the results of GSK3β phosphorylation. Some reduction in hepatic triglyceride level was demonstrated in *S*. *spinosum* treated mice, which lacks statistical significance. H&E staining of the liver demonstrated that *S*. *spinosum* reduced the severity of steatosis. Extensive vacuolation of periacinar hepatocytes was demonstrated in untreated KK-Ay mice, while livers of SSE-treated mice were characterized by a diffuse periacinar, feathery vacuolation, mostly microsteatosis.

### Hepatic gene expression

**Fig 8:**
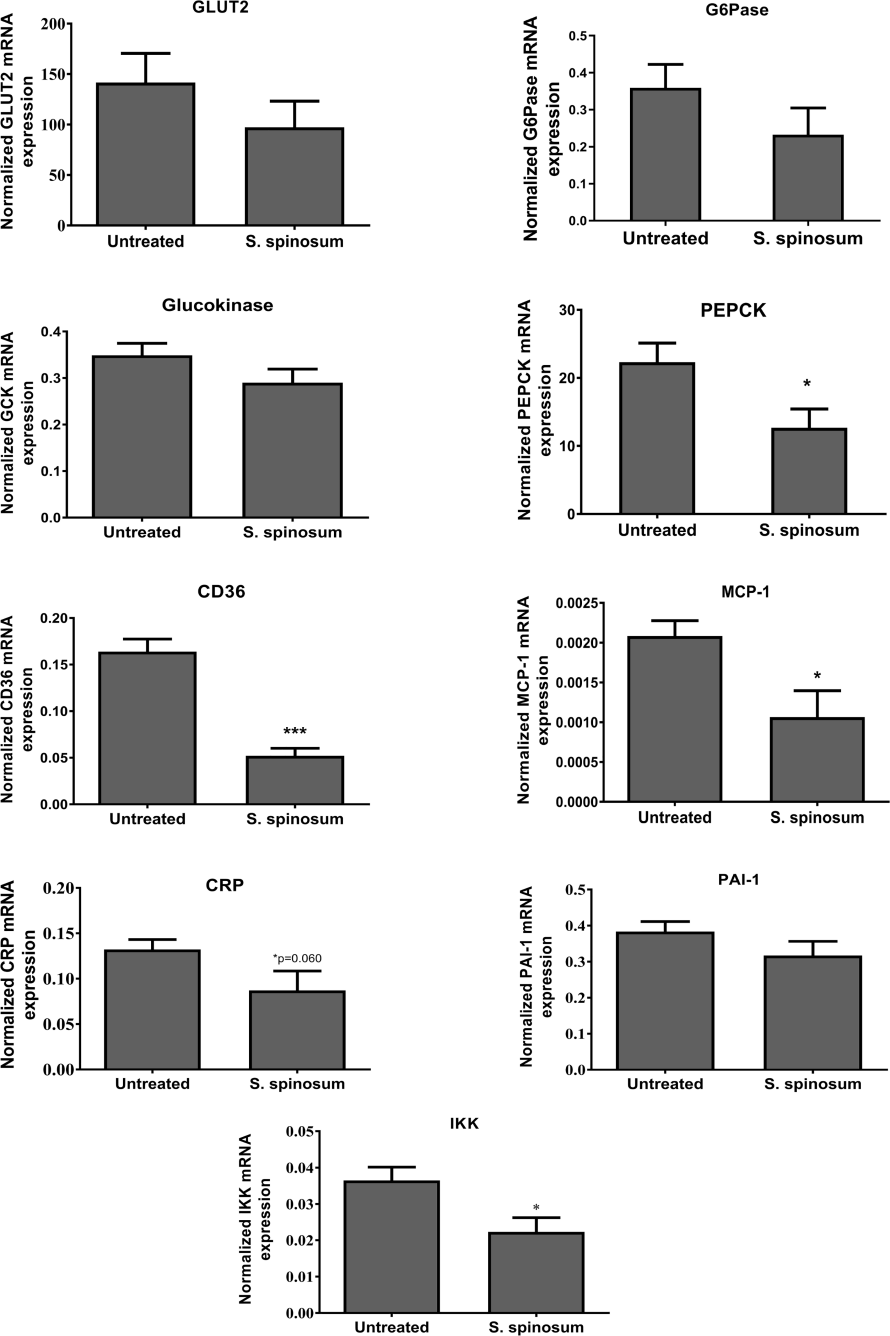
SSE reduced mRNA expression of atherogenic and pro-inflammatory genes. KK-Ay mice were given *S*. *spinosum* extract for 6 weeks as described in methods. Liver was removed at the age of 12 weeks as described in methods. mRNA expression of the indicated genes was measured by real-time PCR. Results were normalized to the expression of housekeeping gene, HPRT. ***P<0.0005 by Student's *t*-test.

In addition, mRNA expression of genes involved in carbohydrates, lipid metabolism, and inflammation was measured (Fig 8). The expression of *PEPCK* was reduced in livers of *S*. *spinosum* treated mice. Both genes encoding for proteins facilitating hepatic glucose transport, *Glut2* and *GCK*, were not affected. Similarly, genes involved in the regulation of lipid metabolism (*PPAR*α, *ACC-1*, *FAS*, *HSL*, *HMGCR*, *SREBP-1C*, and *SREBP2*) were not affected by *S*. *spinosum* treatment in this model (data not shown). *CD36* (fatty acid translocase), which is implicated in lipid metabolism, inflammation, and atherogenesis was significantly reduced in *S*. *spinosum*-treated mice. Similarly, mRNA expression of *MCP-1* and *IKK* pro-inflammatory genes were reduced as well, while *CRP* expression was reduced in a non-significant manner (p = 0.06) and *PAI* expression was not affected.

## Discussion

Insulin resistance of target tissues plays a central role in the pathogenic processes leading to T2D. Initially, the reduced sensitivity to the hormone is compensated by elevated plasma insulin concentration; however, inadequate compensation response, resulting from a progressive worsening of the insulin resistance, or relative failure of insulin-secreting pancreatic-beta cells, causes the disease outbreak [20].

Genetic variation is a significant risk factor for the development of T2D and over 70 genetic loci have been identified so far as associated with the etiology of the disease [21,22]. However, genetic factors by their own cannot explain the sharp climb in the prevalence of T2D over the last decades, suggesting that the radical changes in life habits and other environmental factors are the reason for the outburst of this epidemic [23]. Obesogenic environments include various factors that promote obesity, such as western diet, sedentary lifestyle, exposure to endocrine disruptors, and more [24–28]. While most subjects exposed to an obesogenic environment will experience elevated BMI [29], those carrying the genetic predisposition for diabetes will develop either prediabetes or an overt disease [30].

Obesity is intimately associated with insulin resistance [31]. According to the latest data of the American Centers for Disease Control and Prevention (CDC), from 1988 to 2012 prevalence of diabetes increased significantly over time in all sections of the population and is highly associated with trends of obesity prevalence. In the U.S, over 85% of people with T2DM are overweight or obese [32]. The metabolic overload, which is a characterizing feature of obesity, is recognized as inducing insulin resistance via several mechanisms, such as the promotion of low-grade chronic inflammation, alteration in adipose tissue function, oxidative stress, and mitochondrial dysfunction [33,34]. These pathological mechanisms are not necessarily identical to ones associated with genetic aberration, leading to insulin resistance. Thus, as obesogenic environment and genetic predisposition activate various mechanisms to induce the pathology of T2DM, antidiabetic treatment should be evaluated according to its efficacy to improve glucose tolerance that develops through both etiologies. Thus, in addition to our previous studies demonstrating the glucose-lowering properties of *S*. *spinosum* extract in a genetic mice model of the disease [15,16], it is important to validate effectiveness of this extract in mice developing glucose intolerance due to environmental-related etiology.

In this study, we show that SSE exerts glucose lowering effects on HFD-fed C57bl/6 mice, a model of diet-induced glucose intolerance with the absence of a significant genetic predisposition to the disease. We also further investigated the effects of SSE on insulin sensitivity in KK-Ay mice, developing T2D on a genetic background. Improved glucose tolerance and reduced insulin resistance were demonstrated in HFD-fed mice given *S*. *spinosum*, as we also previously found in the genetic model of T2D, with the KK-Ay mice [15,16]. The improved sensitivity to the hormone was validated by lower fasting serum insulin and higher activation of the insulin signaling cascade in skeletal muscle and liver of treated mice in both models.

Skeletal muscle and liver play a central role in maintaining whole body glucose homeostasis. The first is considered to be responsible for >75% of insulin-induced glucose disposal from blood under either euglycemia or hyperglycemia [35], while the second is the organ enabling de-novo glucose production. Under physiological conditions, insulin activates signaling pathways leading to increased glucose uptake to target tissues, activation of anabolic pathways enabling glycogen synthesis in liver and muscles, and inhibition of hepatic gluconeogenesis. Thus, hyperglycemia associated with disturbances in insulin action results from both low glucose disposal and unrepressed glucose production. Our results, demonstrating that SSE increased the phosphorylation of key signaling proteins in skeletal muscle and liver, suggest that both functions–glucose uptake and glucose production–are better regulated in *S*. *spinosum* treated mice.

Insulin failed to phosphorylate IR, PKB, and GSK3β in KK-Ay, reflecting the severe insulin resistance of these mice. SSE reduced this resistance, enabling the phosphorylation of these proteins in skeletal muscle. In the liver, *S*. *spinosum* increased both basal and insulin-induced IR phosphorylation, increased insulin-induced PKB phosphorylation, while GSK3β was not affected in this tissue. These results suggest that although *S*. *spinosum* beneficially affects both tissues, there are some tissue specific effects of the extract.

Fatty liver is associated with T2D, considered to be the hepatic manifestation of the metabolic syndrome [36]. Lower severity of steatosis was found in *S*. *spinosum*-treated KK-Ay mice, accompanied with lower mRNA expression of CD36, a biomarker of atherogenesis and metabolic dysfunction as well as some others pro-inflammatory genes. This suggests that in addition to its effect on glucose tolerance, SSE might be beneficial to improve additional manifestations of the metabolic syndrome, such as atherosclerosis, dyslipidemia, and inflammation. These potential effects are currently under investigation using appropriate models for these diseases.

The severity of insulin resistance is lower in HFD-fed mice compared to KK-Ay mice. The fasting level of serum insulin is high, indicating that insulin sensitivity is defective. However, glucose tolerance tests resemble the state of prediabetes, in which fasting glucose level is only moderately increased, while the glucose disposal rate is slower than normal. Transmission of insulin signaling is aberrant in HFD-fed mice, especially in the muscle, supporting the development of insulin resistance, although the severity of the resistance is lower than is found in KK-Ay mice. Our results demonstrated that SSE increased insulin-induced phosphorylation of IR, PKB and PRAS40. This signaling cascade is involved in the induction of glucose transport, and might mediate the improved glucose tolerance of SSE-treated mice [37]. Surprisingly, insulin-induced phosphorylation of GSK3β was not detected in muscle of either STD or HFD-fed mice, this might be because this downstream event requires longer time of insulin stimulation than done in this set of experiments.

Interestingly, basal phosphorylation of IR and GSK is elevated in the liver of HFD-fed mice. It can thus be suggested that hypersecretion of insulin partially compensates for the developing hepatic insulin resistance. However, as these HFD-fed mice experienced oversupply of nutrients, there is a requirement for higher activation of an anabolic cascade in order to overcome the overload of energy and successfully store the excess nutrients. It was previously reported that energy excess accompanying HFD induces an increase in hepatic glycogen, however, there is an upper limit to this energy store, and upon reaching it, energy storage is shifted from glycogen to lipid, leading to hepatic steatosis [38]. In line with these observations, we found that SSE extract increased hepatic glycogen storage capacity and reduced steatosis in HFD-fed mice. Elevation in mRNA expression of GCK, a gene encoding for an enzyme that facilitates hepatic glucose transport, also supports the hypothesis that *S*. *spinosum* increased glucose disposal from blood to be stored as glycogen in the liver. It should also be noted that while hepatic *CD36* gene expression was not affected by *S*. *spinosum* extract, *AdipoR2* mRNA expression was increased. Adiponectin is known to decrease hepatic insulin resistance and to attenuate liver inflammation and fibrosis through binding to AdipoR1 and AdipoR2, the latter is preferentially expressed in rodents livers [39,40]. The increase in the expression of this receptor supports the insulin sensitizing effects of SSE.

Hyperinsulinemia is considered to be not only a consequence of insulin resistance, but also a contributing factor for this disturbance, especially in the presence of high free fatty acids [41]. This vicious cycle is suggested to be the result of a negative feedback loop, activated as a result of high serum insulin [42]. In addition to worsening the pathology of insulin resistance, hyperinsulinemia has negative effects on pancreatic β cells by enhancing oxidative stress and cell damage [43]. Thus, correction of hyperinsulinemia is an important target of insulin sensitizer agents. Our results, demonstrating a significant reduction in blood insulin in *S*. *spinosum* treated HFD-fed mice and also in KK-Ay mice [15], support the beneficial antidiabetic potential of this herbal preparation.

HFD-fed mice develop hepatic steatosis. This pathology is of lower severity on the spectrum of NAFLD, and is characterized by the accumulation of lipids in the liver, but the absence of hepatic inflammation, fibrosis, and failure. Although found to enhance the activation of insulin signaling in the liver, *S*. *spinosum* did not reduce hepatic lipid accumulation in this model. Interestingly, while genes involved in lipid metabolism were downregulated by HFD, as was also previously reported [44,45], this was not seen in HFD-fed mice administrated with SSE, demonstrating a gene expression profile which is similar to that of STD-fed mice. The metabolic outcomes of this observation are unclear, and it should be further clarified whether the normalization of the gene expression profile has positive or negative effects on whole-body lipid homeostasis and specifically on hepatic function. The effects of SSE on blood lipids and the development of atherosclerosis are currently under investigation using appropriate models, such as the ApoE KO mice. In addition, a mice model of steatohepatitis is utilized in order to further clarify the effects of the extract on NAFLD.

Various disturbances (e.g. inflammation, endothelial dysfunction, and dyslipidemia) accompanying the metabolic syndrome share a common pathology of insulin resistance [46]. Our study, demonstrating that *S*. *spinosum* improved insulin sensitivity in two models of insulin resistance, supports the need to perform additional research in order to clarify the effects of this extract on other manifestations of the metabolic syndrome.

## Supporting information

S1 Fig Original blots presented in Fig 2. A. Original blots presented in Fig 2A. When a significant different in the molecular weight of protein of interest exists, some of the membranes were re-blotted with additional primary antibodies, thus the "non-specific bands" are the bands developed as a result of the previous primary antibody still exists, as is seen in the blot of pPKB and pIR.
(DOCX)

S2 Fig Original blots presented in Fig 3. A. original blots presented in Fig 3A. When a significant different in the molecular weight of protein of interest exists, some of the membranes were re-blotted with additional primary antibodies, thus the "non-specific bands" are the bands developed as a result of the previous primary antibody still exists, as is seen in the blot of pPKB.
(DOCX)

S3 Fig Original blots presented in Fig 6. A. Original blots presented in Fig 6A. B. Original blots presented in Fig 6B. In this set of blots, the order of loading was as followed: *S*. *spinosum* treated without insulin stimulation (3 mice), S. spinosum treated with insulin stimulation (3 mice), control mice without insulin stimulation (4 mice), control mice with insulin stimulation (4 mice). In order to present the results in more logical way, in Fig 5A and 5B the control bands were separated from *S*. *spinosum* bands and are presented at the left of the panel, without any manipulation of the results.
(DOCX)
