# Supplementary material for: Sarcopoterium spinosum extract improved insulin sensitivity in mice models of glucose intolerance and diabetes
Source: PLoS One. 2018 May 16;13(5):e0196736. doi: 10.1371/journal.pone.0196736 (PMC5955592; doi:10.1371/journal.pone.0196736)
Supplement: S1 Fig — A. Original blots presented in Fig 2A. When a significant different in the molecular weight of protein of interest exists, some of the membranes were re-blotted with additional primary antibodies, thus the "non-specific bands" are the bands developed as a result of the previous primary antibody still exists, as is seen in the blot of pPKB and pIR. (DOCX) [file pone.0196736.s001.docx]

Supplementary figure 1

| muscle |  | Chemiluminescence | Merge (Chemiluminescence+ bright field) |
| --- | --- | --- | --- |
| pIR | STD | 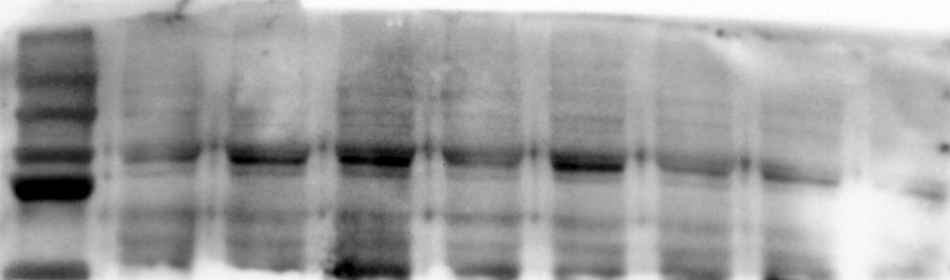  75kD  100kD | 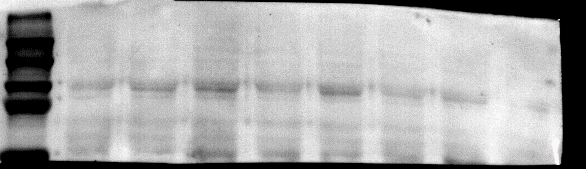 |
|  | HFD | 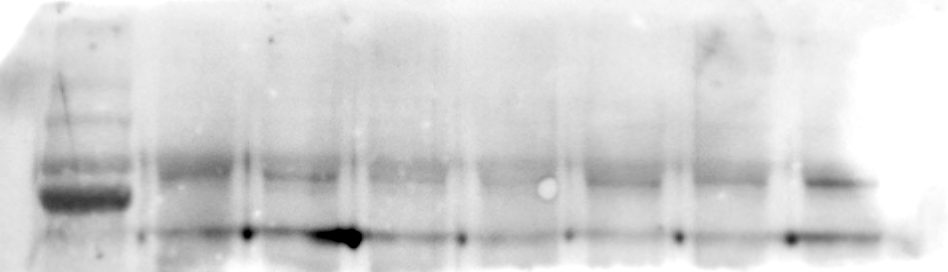  75kD  100kD | 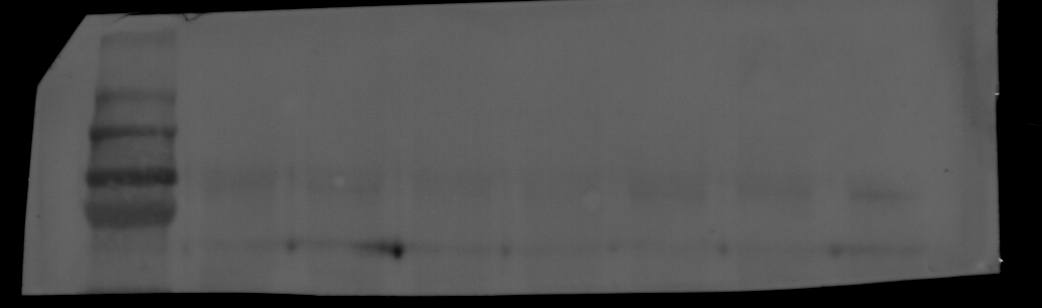 |
|  | HFD.S | 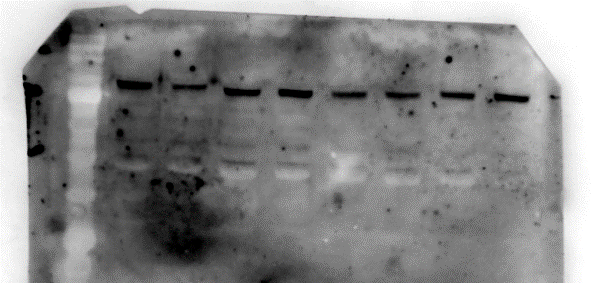 | 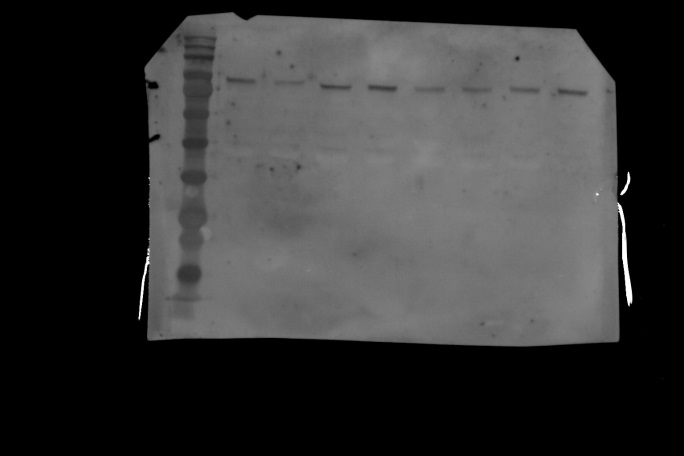  75kD  100kD |
| IR | STD | 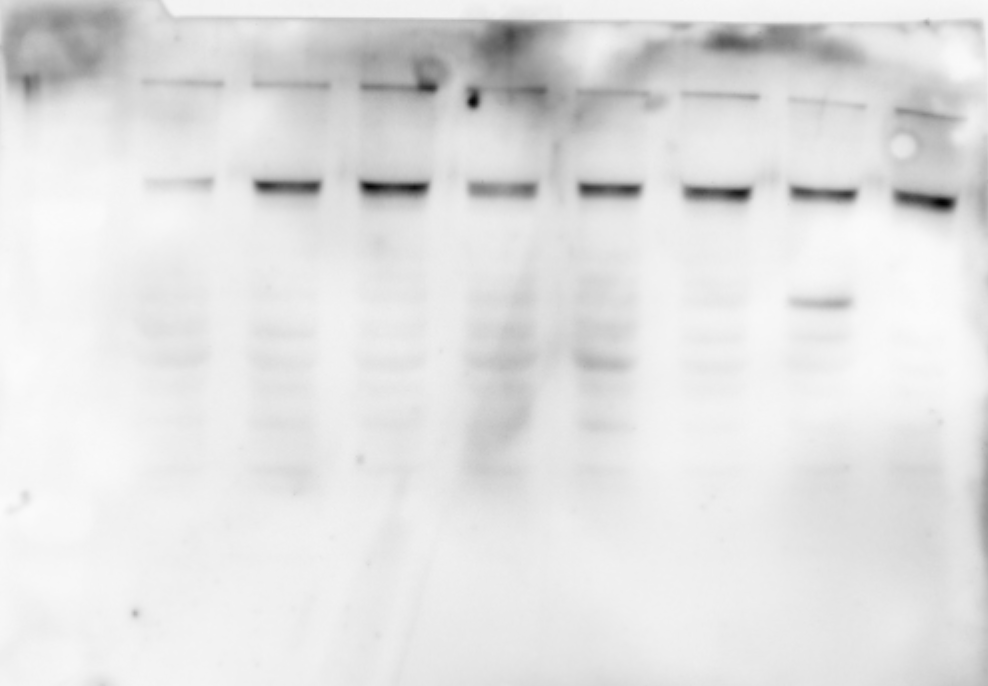 | 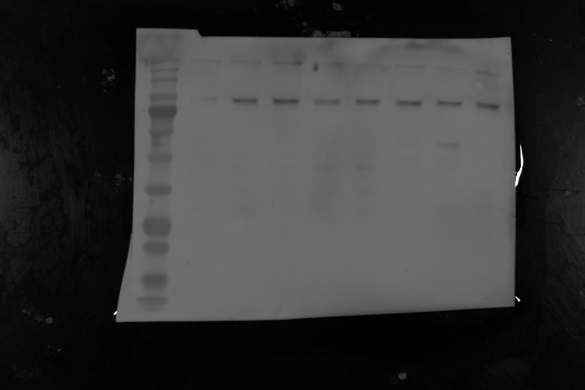  75kD  100kD |
|  | HFD | 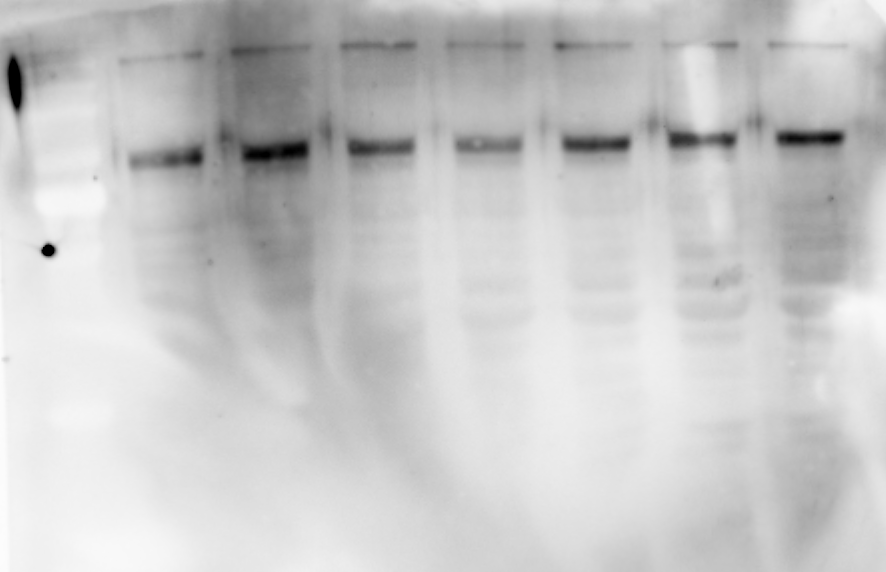 | 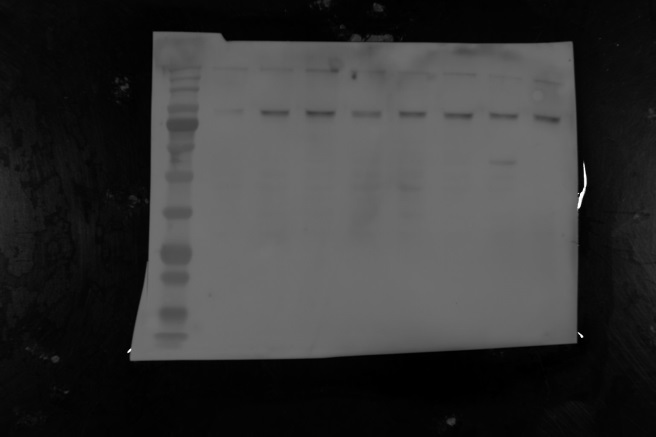  75kD  100kD |
|  | HFD.S | 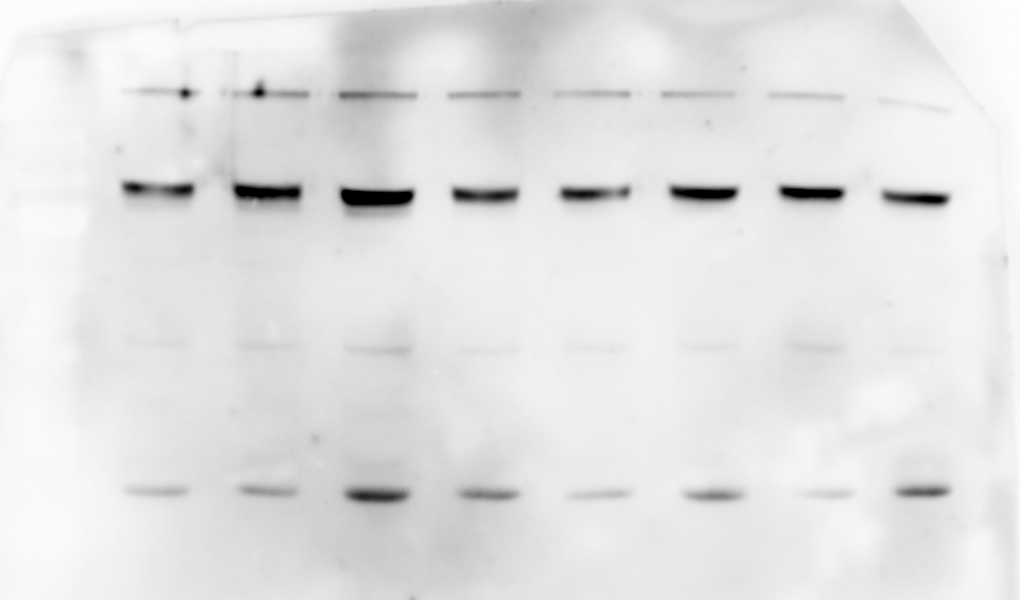 | 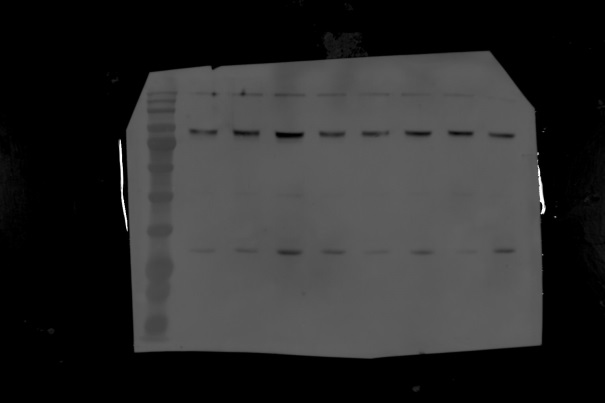  75kD  100kD |
| pPKB | STD | 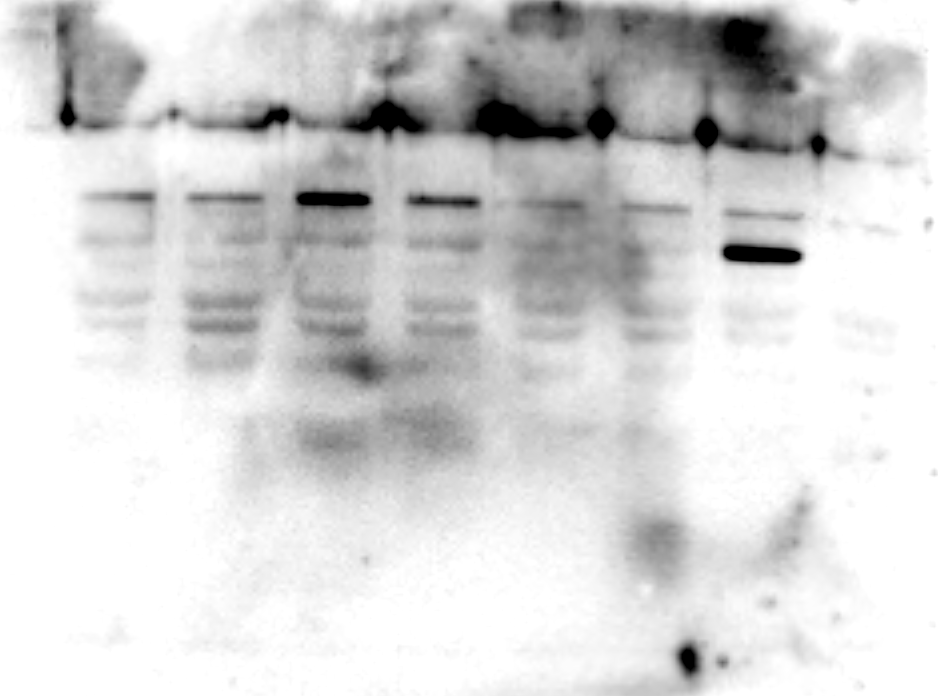 | 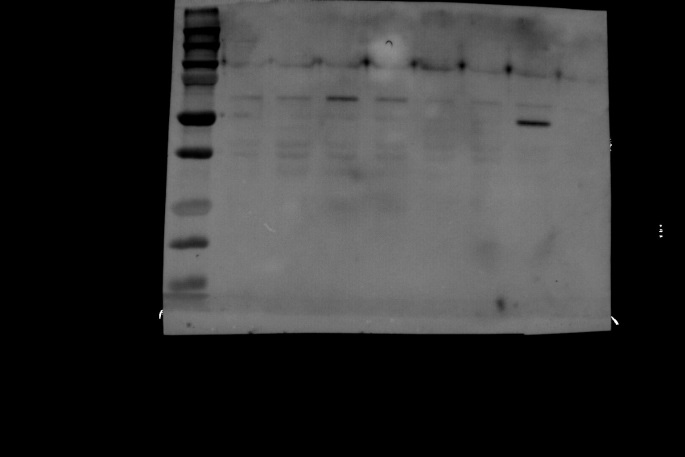  50kD  75kD |
|  | HFD | 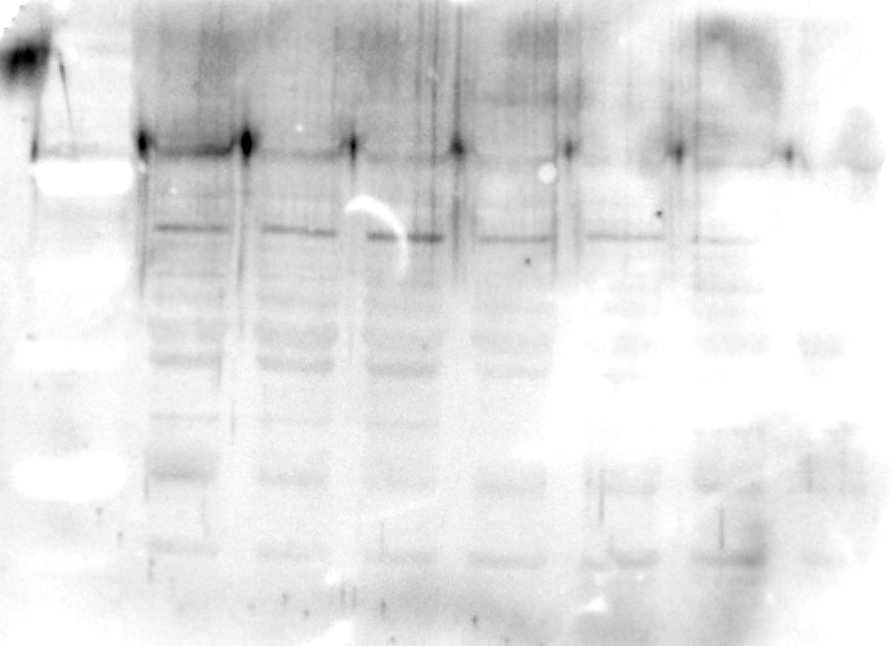 | 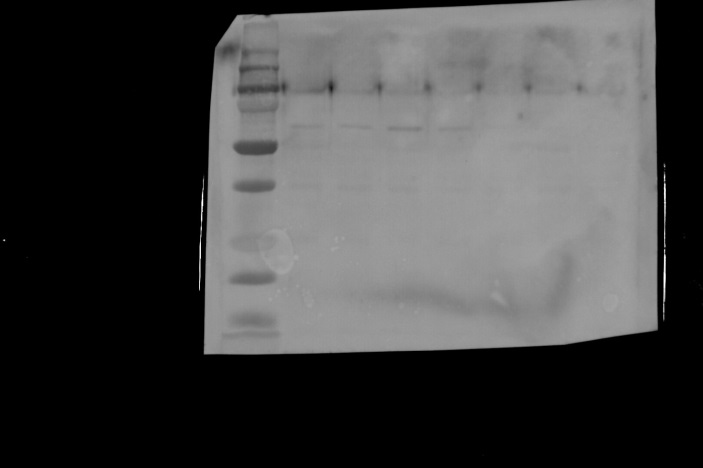  50kD  75kD |
|  | HFD.S | 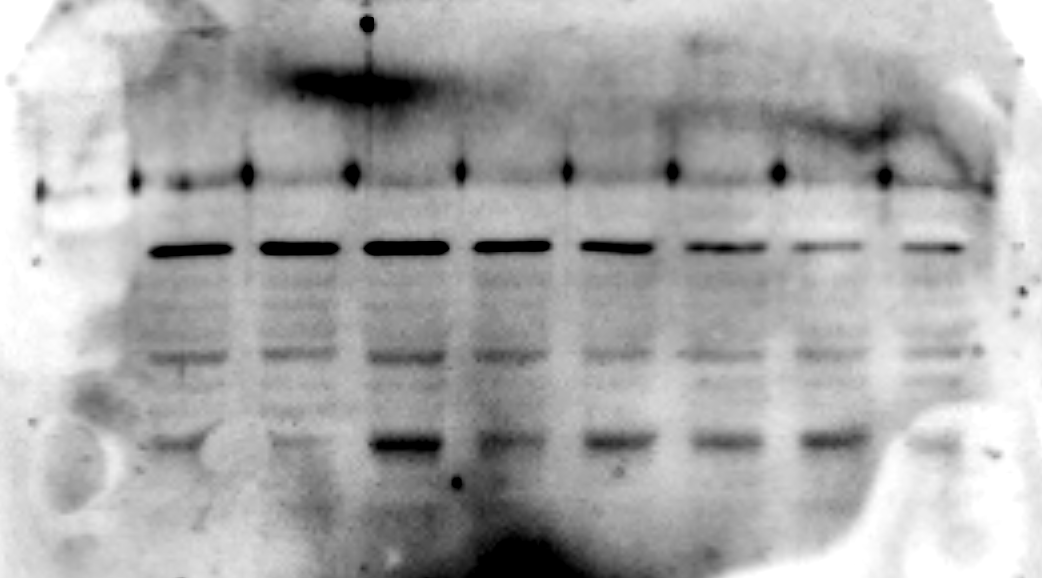 | 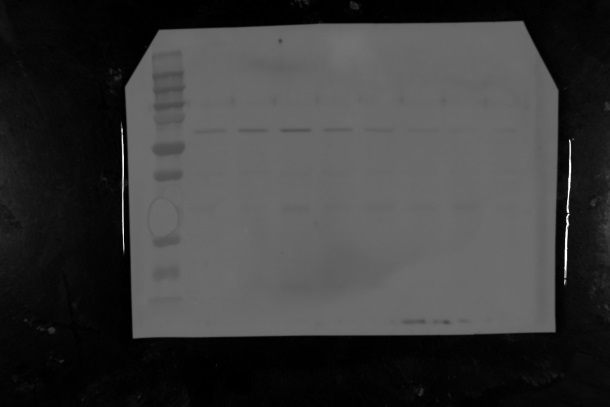  50kD  75kD |
| PKB | STD | 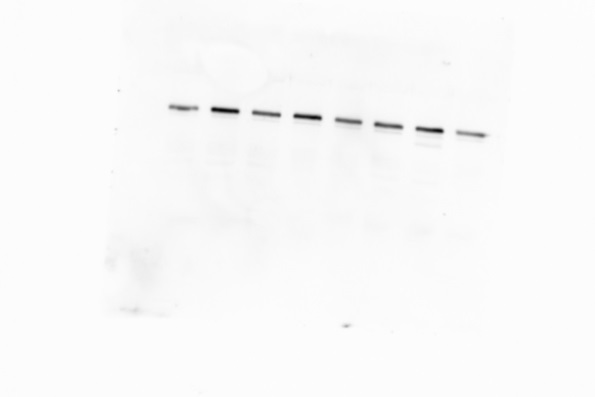 | 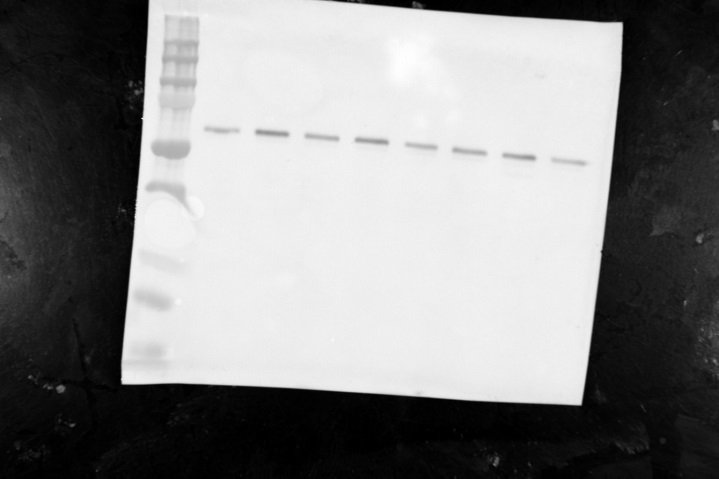  50kD  75kD |
|  | HFD | 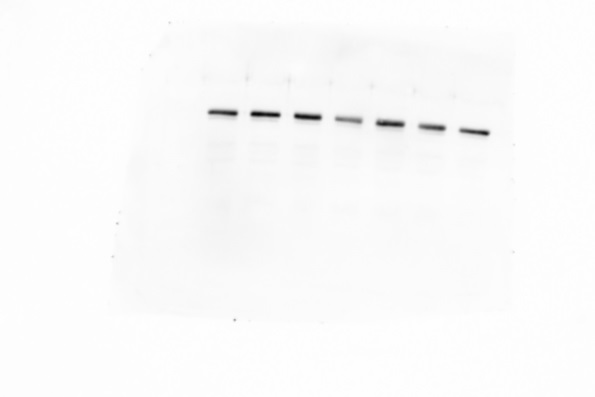 | 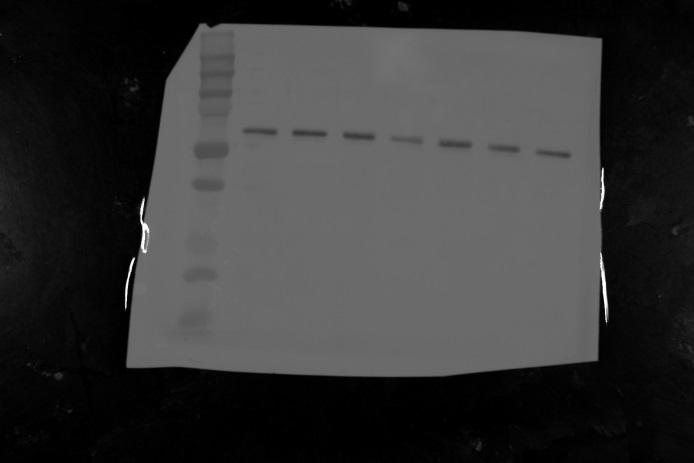  50kD  75kD |
|  | HFD.S | 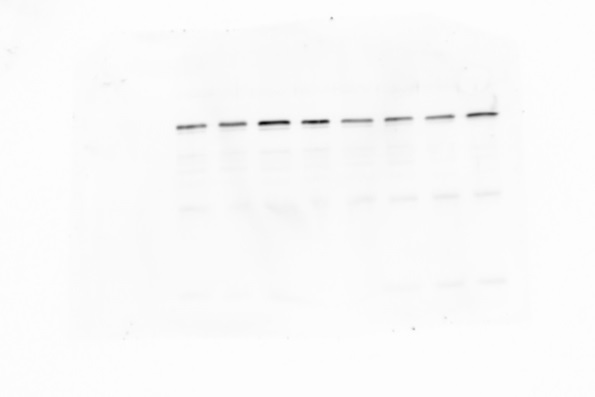 | 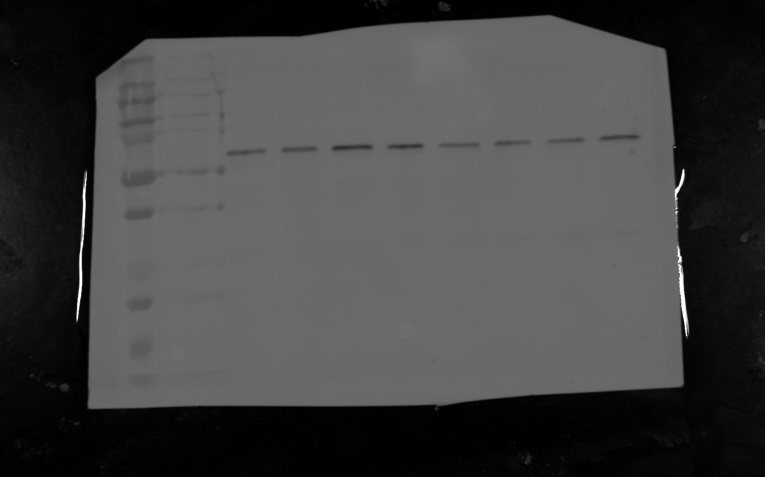  75kD  50kD |
| pGSK | STD | 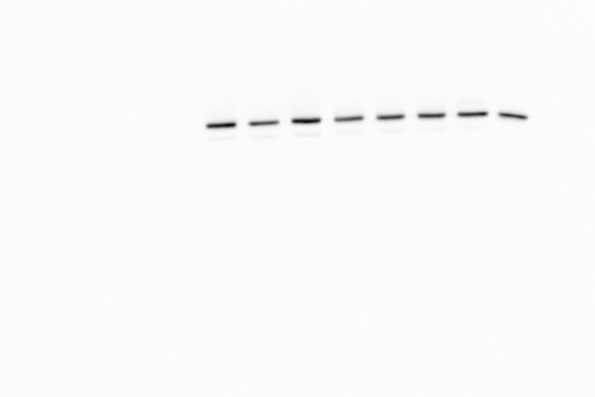 | 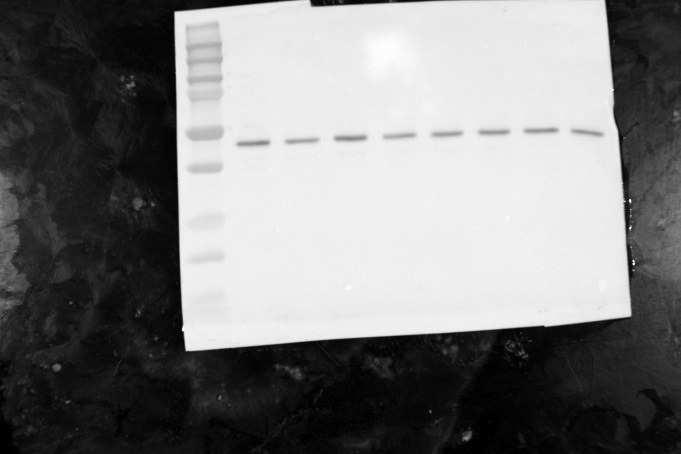  37kD  50kD |
|  | HFD | 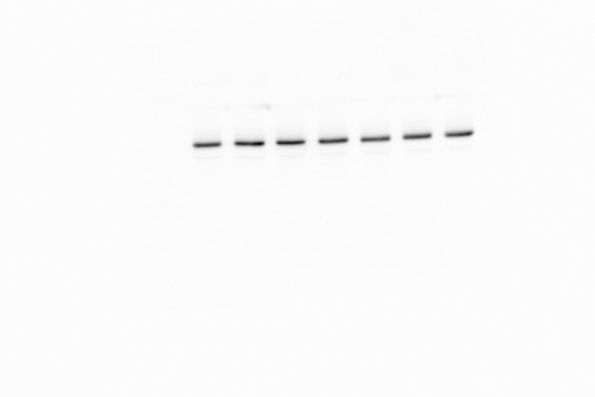 | 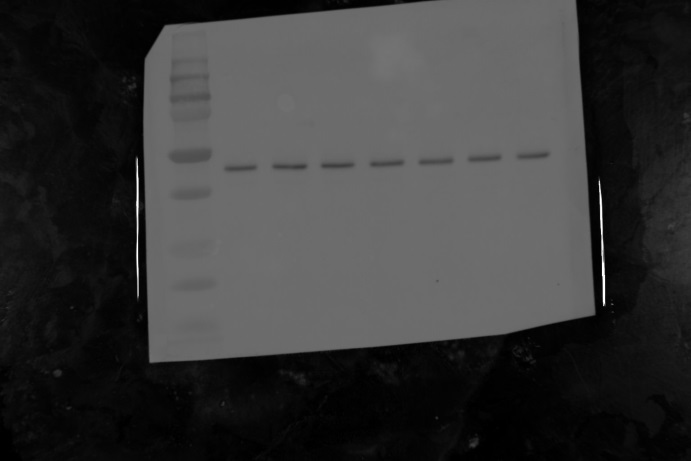  37kD  50kD |
|  | HFD.S | 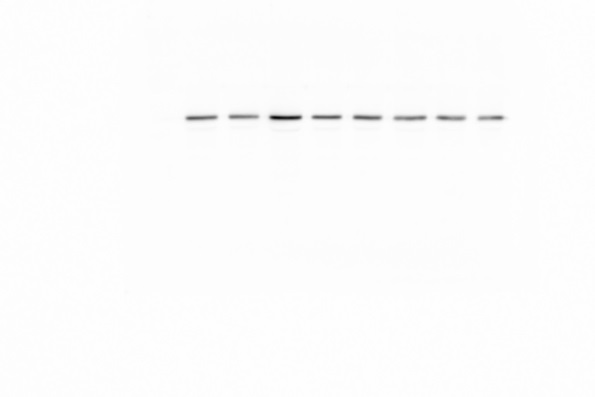 | 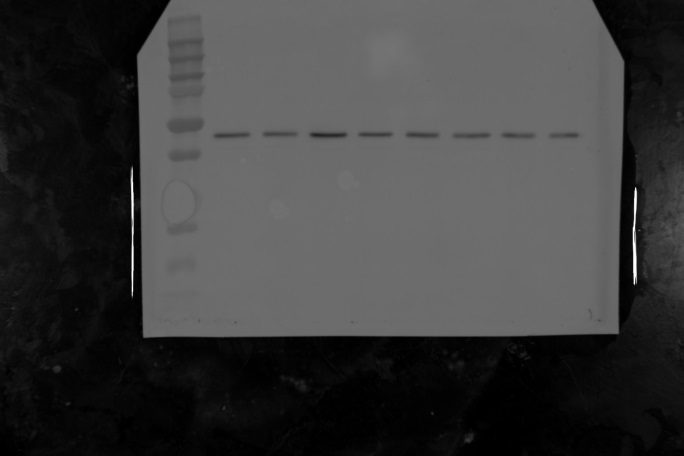  37kD  50kD |
| GSK | STD | 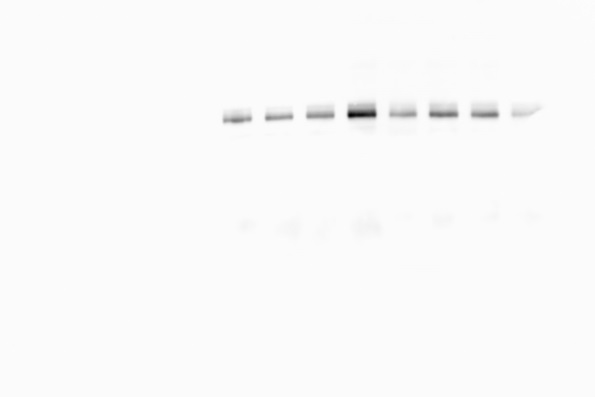 | 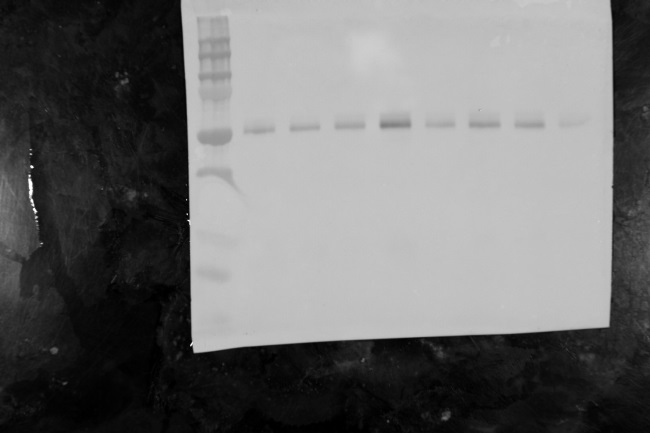  37kD  50kD |
|  | HFD | 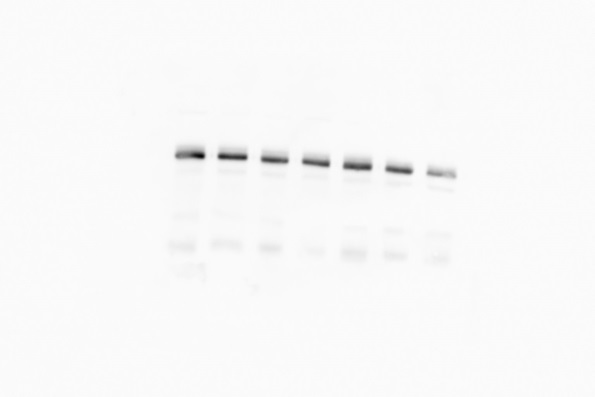 | 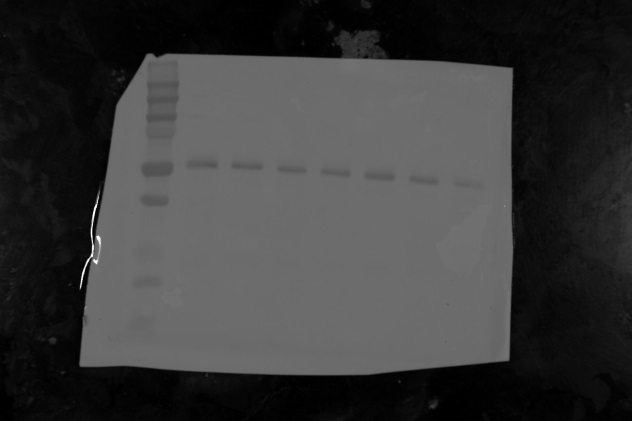  37kD  50kD |
|  | HFD.S | 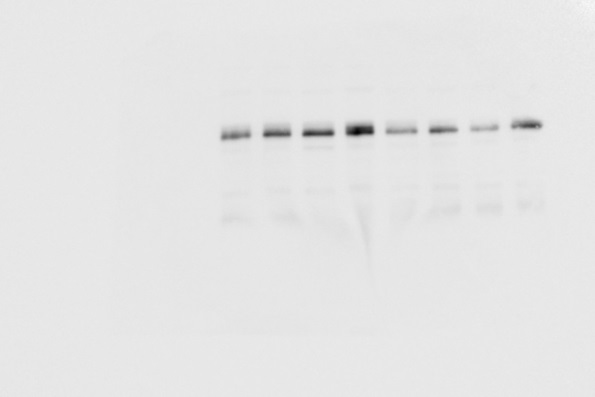 | 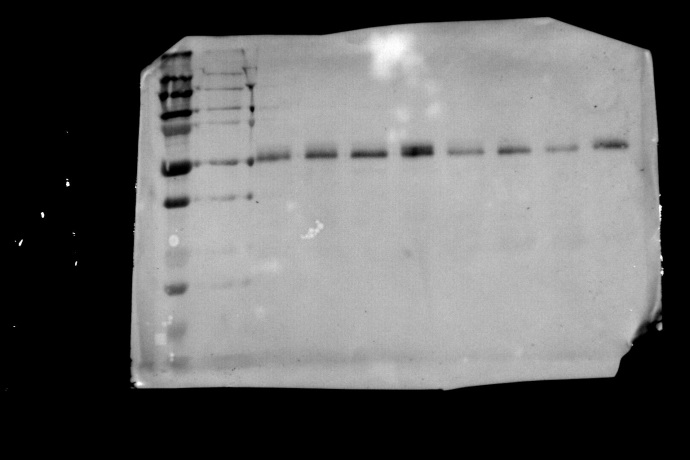  37kD  50kD |
| pPRAS | STD | 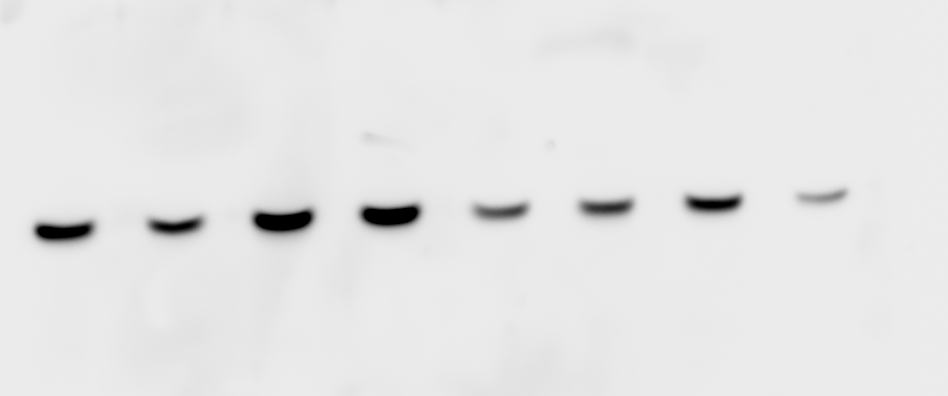 | 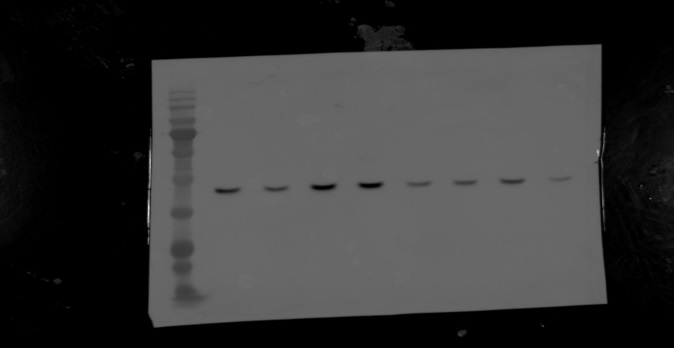  35kD  45kD |
|  | HFD | 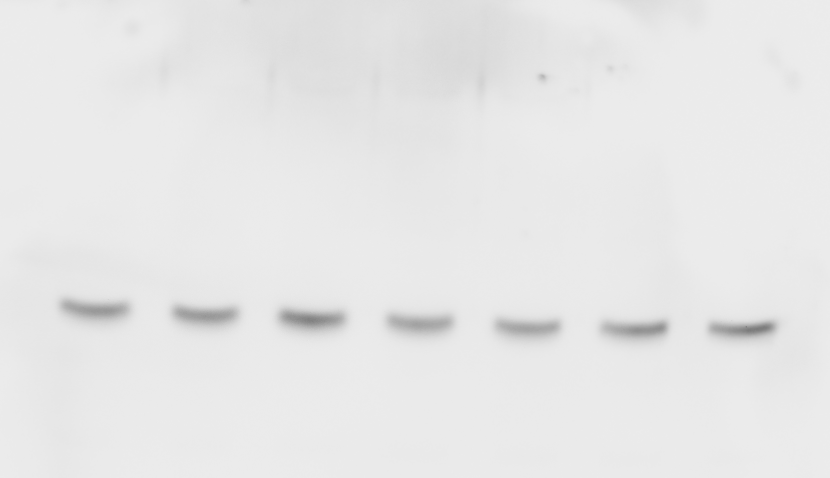 | 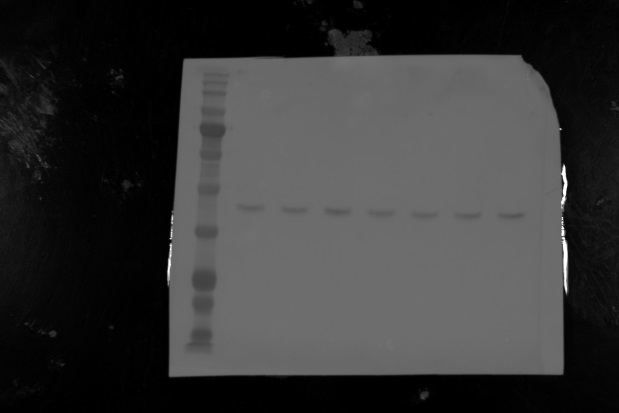  35kD  45kD |
|  | HFD.S | 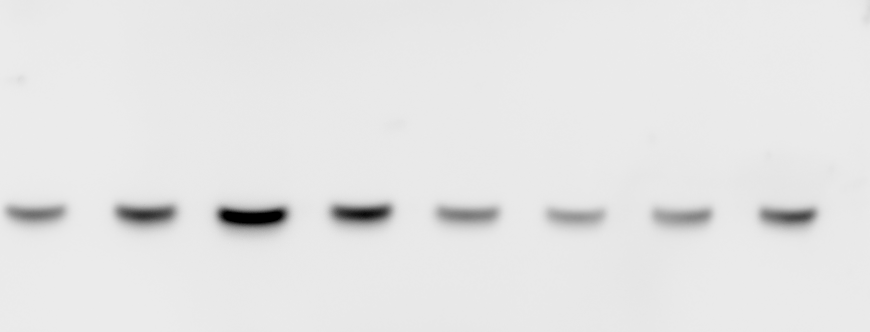 | 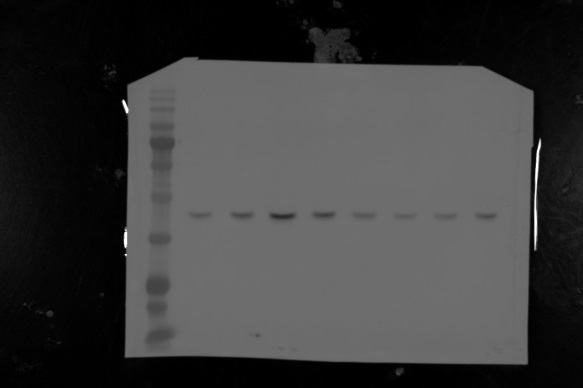  35kD  45kD |
| ACTIN | STD | 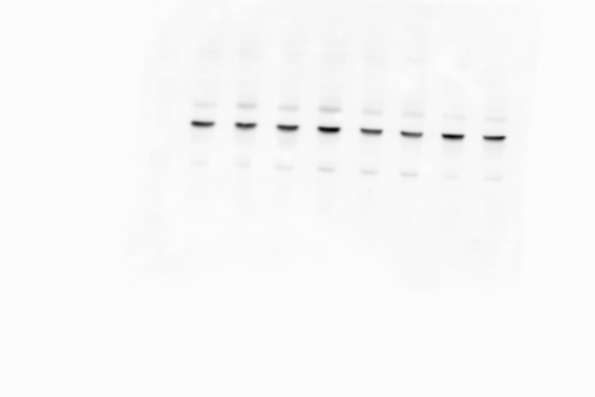 | 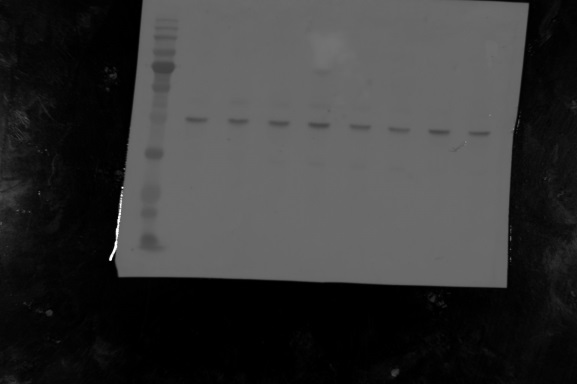  35kD  45kD |
|  | HFD | 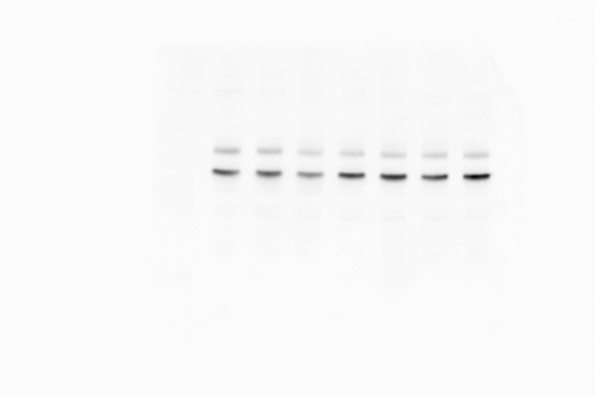 | 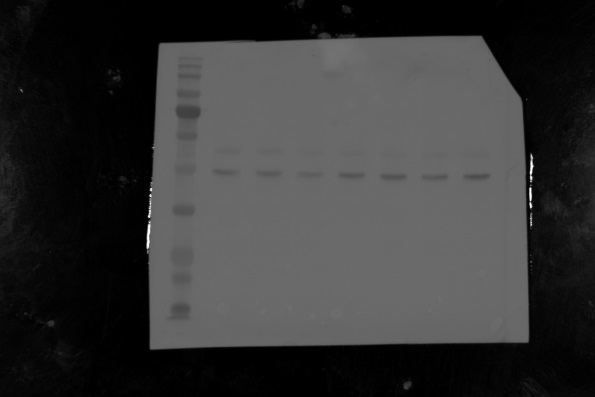  35kD  45kD |
|  | HFD.S | 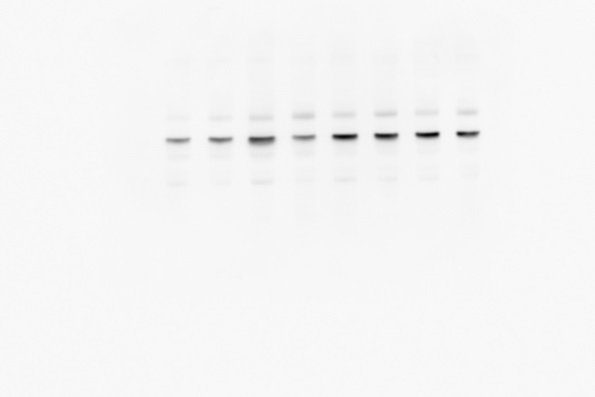 | 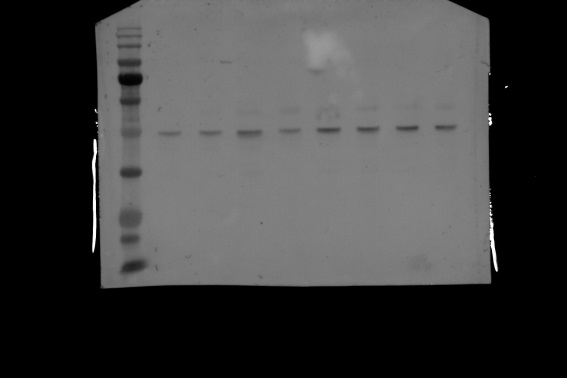  45kD  35kD |

Supplementary figure 1. Original blots presented in Figure 2. When a significant different in the molecular weight of protein of interest exists, some of the membranes were re-blotted with additional primary antibodies, thus the "non-specific bands" are the bands developed as a result of the previous primary antibody still exists, as is seen in the blot of pPKB and pIR.
