# Supplementary material for: Sarcopoterium spinosum extract improved insulin sensitivity in mice models of glucose intolerance and diabetes
Source: PLoS One. 2018 May 16;13(5):e0196736. doi: 10.1371/journal.pone.0196736 (PMC5955592; doi:10.1371/journal.pone.0196736)
Supplement: S2 Fig — A. original blots presented in Fig 3A. When a significant different in the molecular weight of protein of interest exists, some of the membranes were re-blotted with additional primary antibodies, thus the "non-specific bands" are the bands developed as a result of the previous primary antibody still exists, as is seen in the blot of pPKB. (DOCX) [file pone.0196736.s002.docx]

Supplementary figure 2

| Liver |  | Chemiluminescence | Merge (Chemiluminescence+ bright field) |
| --- | --- | --- | --- |
| pIR | STD | 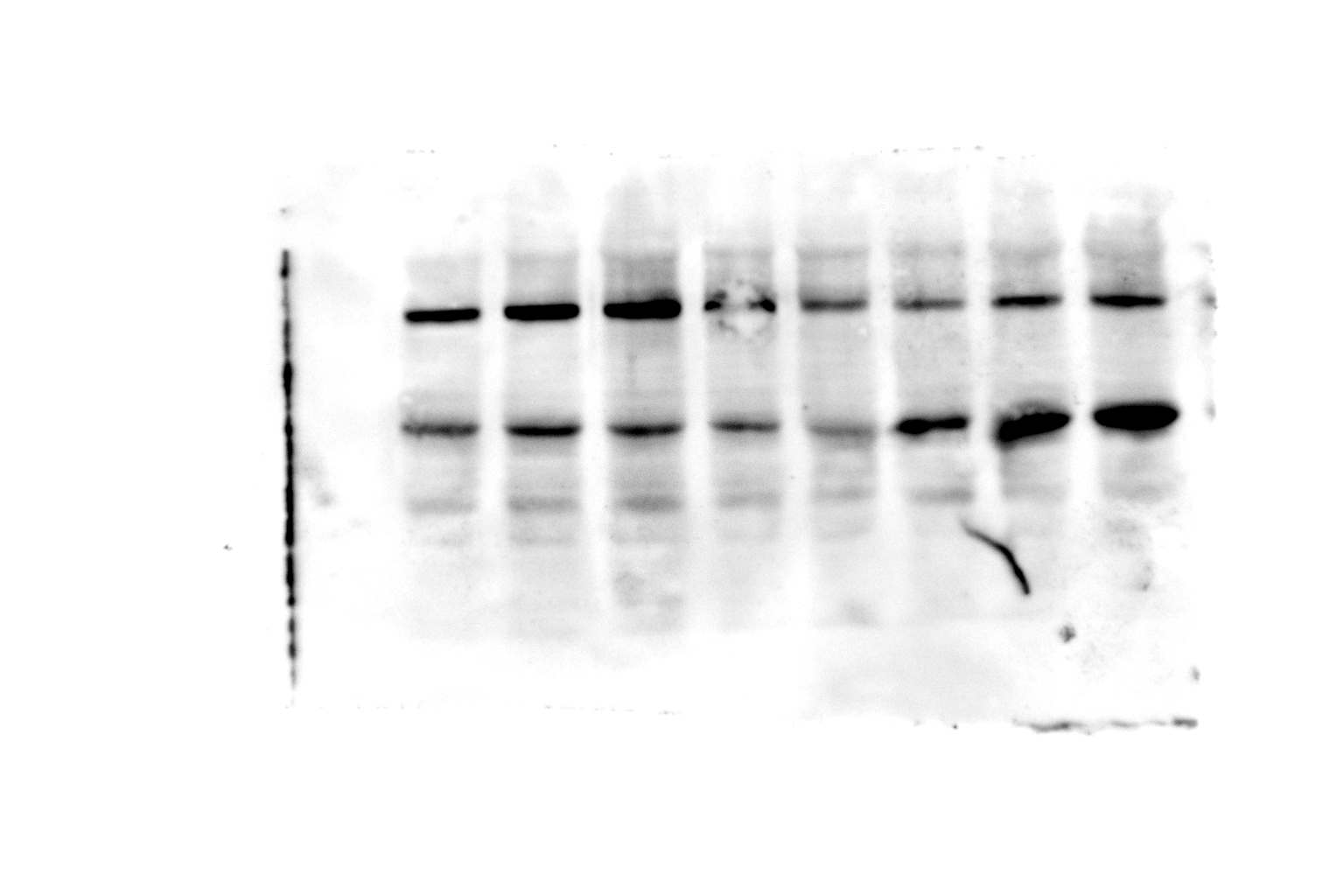  75kD  100kD | 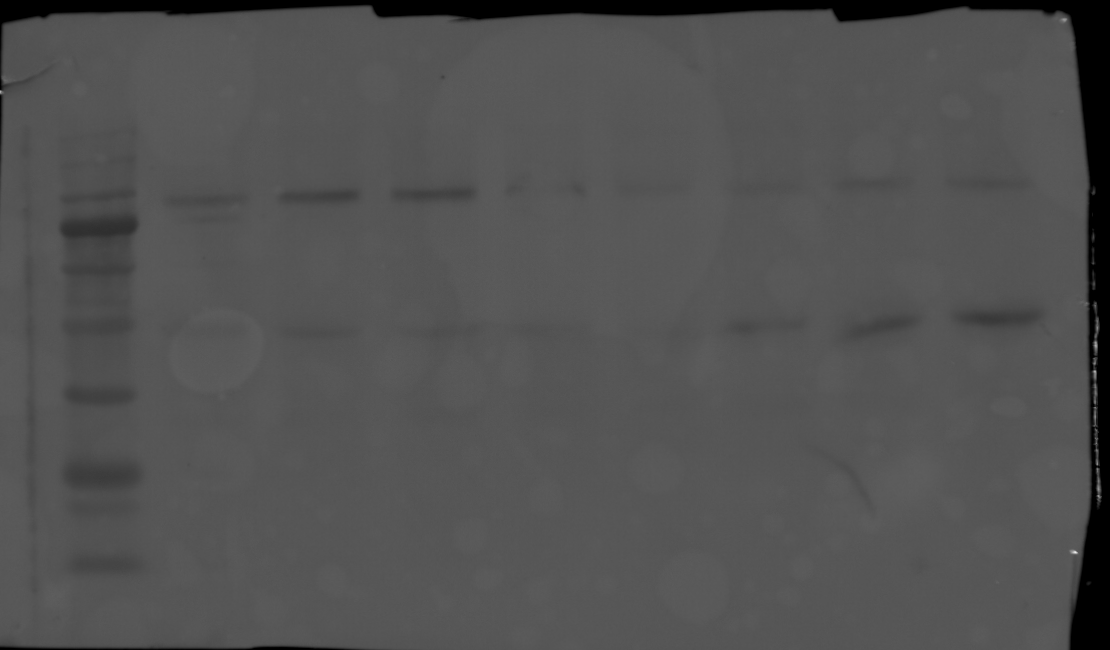 |
|  | HFD | 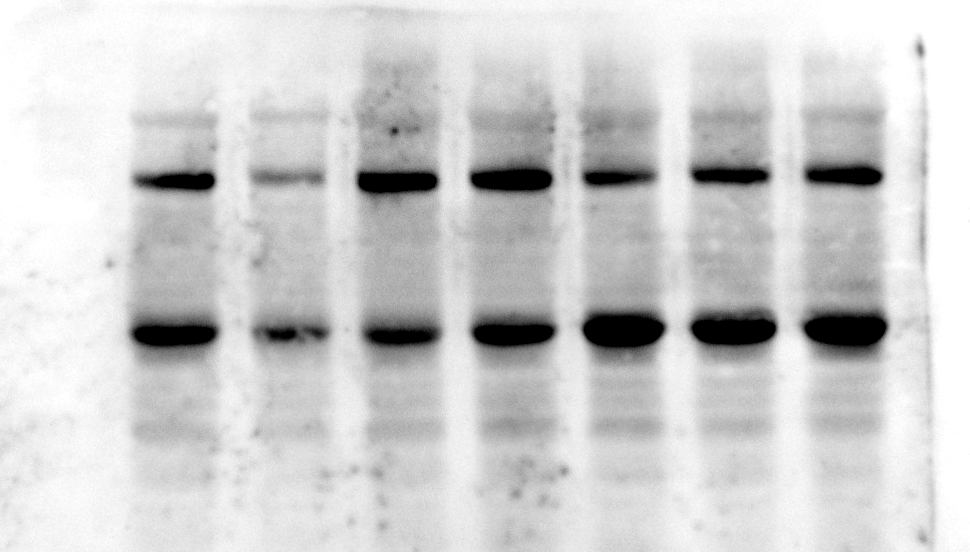 | 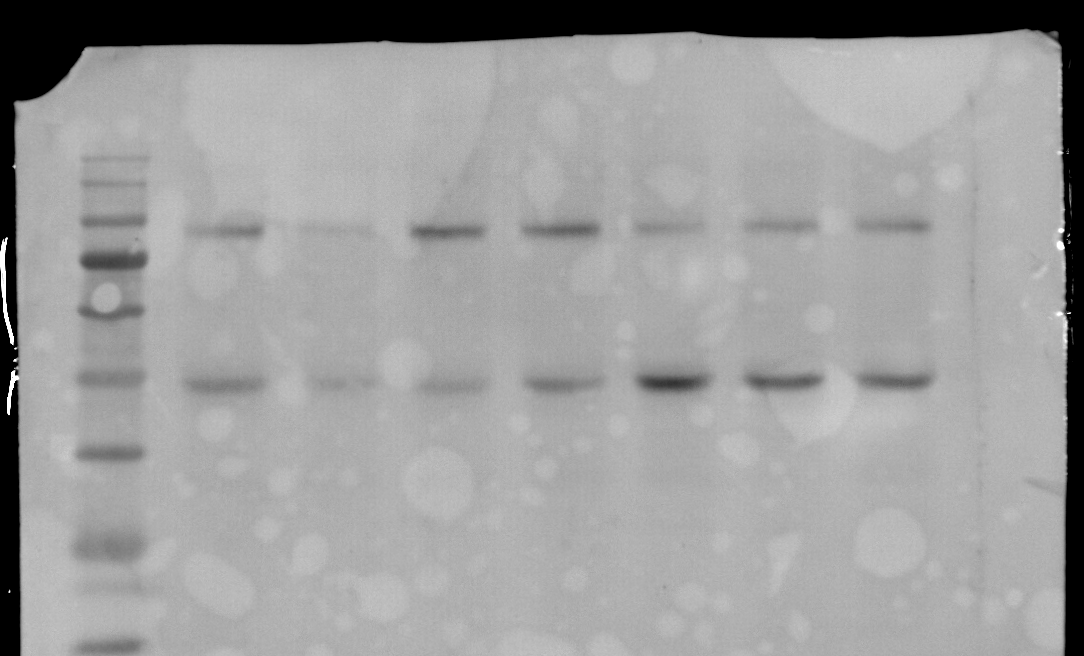  75kD  100kD |
|  | HFD.S | 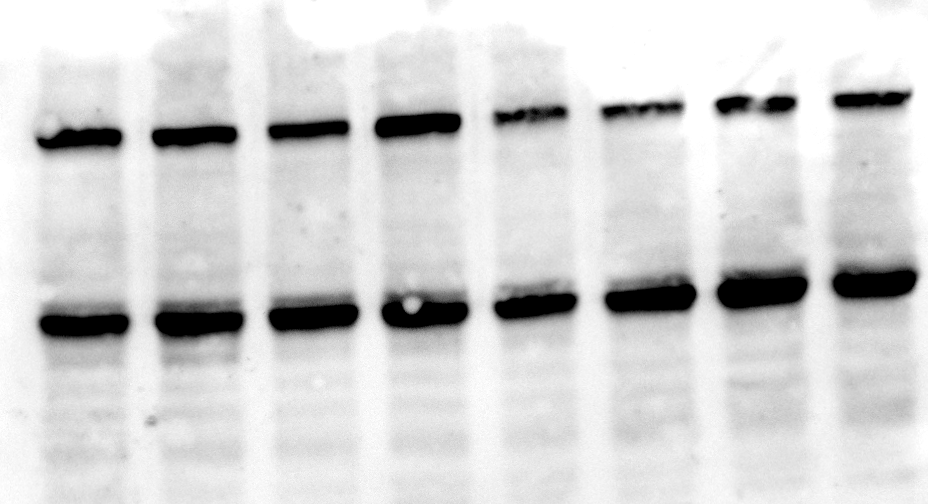 | 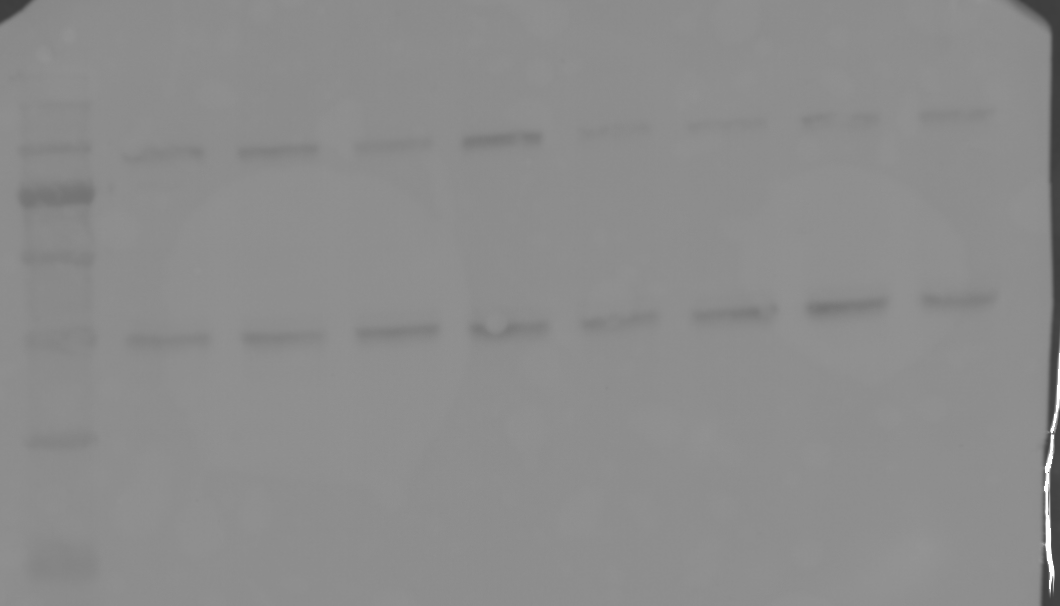  75kD  100kD |
| IR | STD | 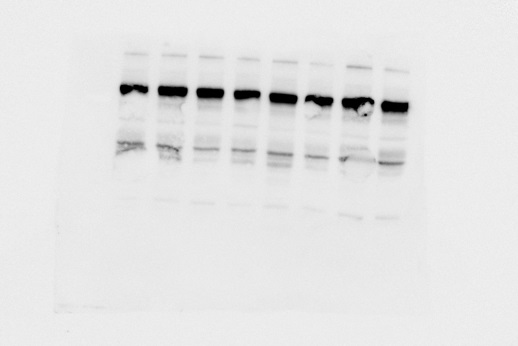 | 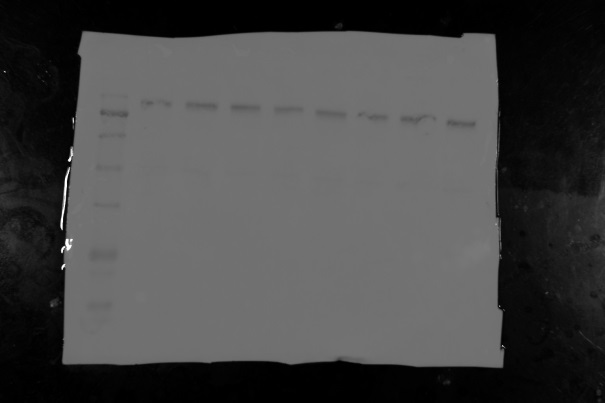  75kD  100kD |
|  | HFD | 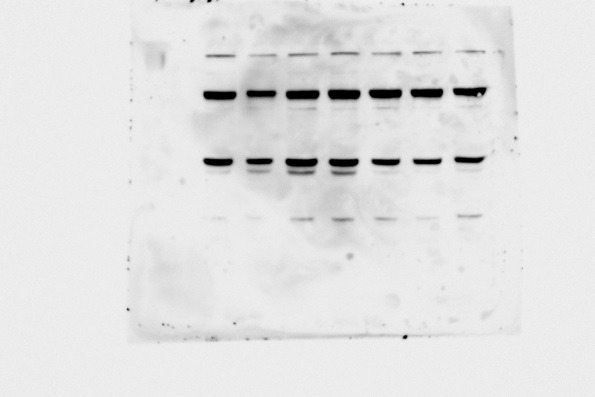 | 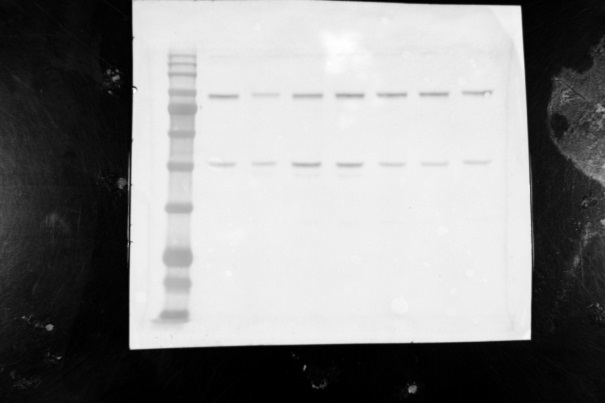  75kD  100kD |
|  | HFD.S | 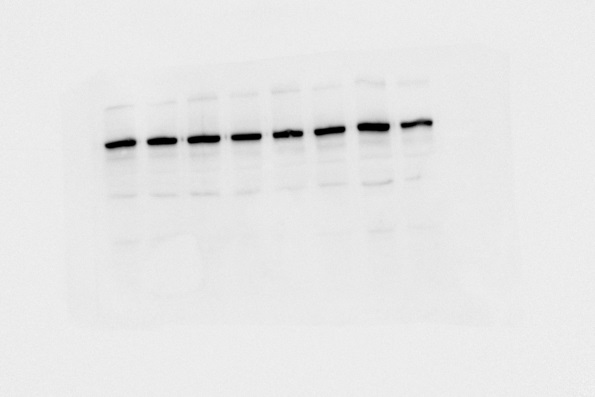 | 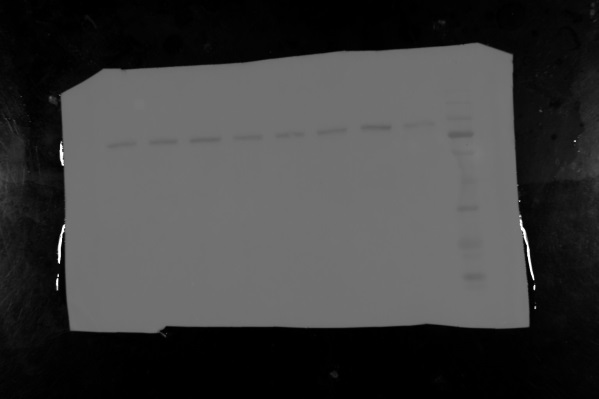  75kD  100kD |
| pPKB | STD | 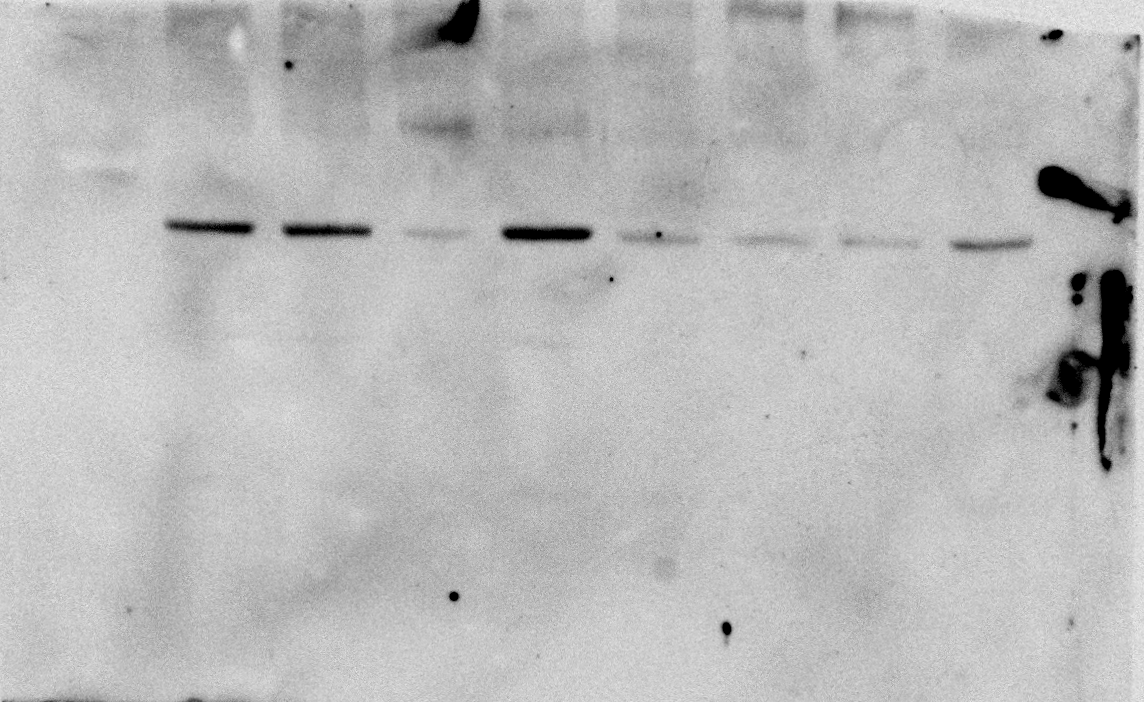 | 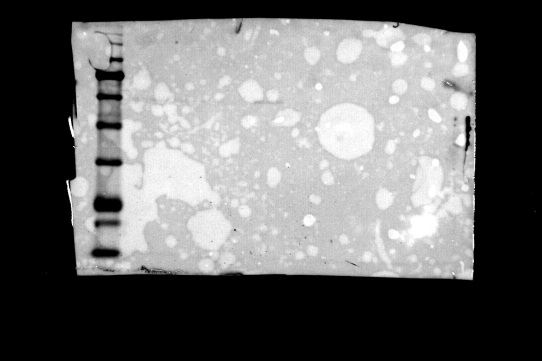  45kD  63kD |
|  | HFD | 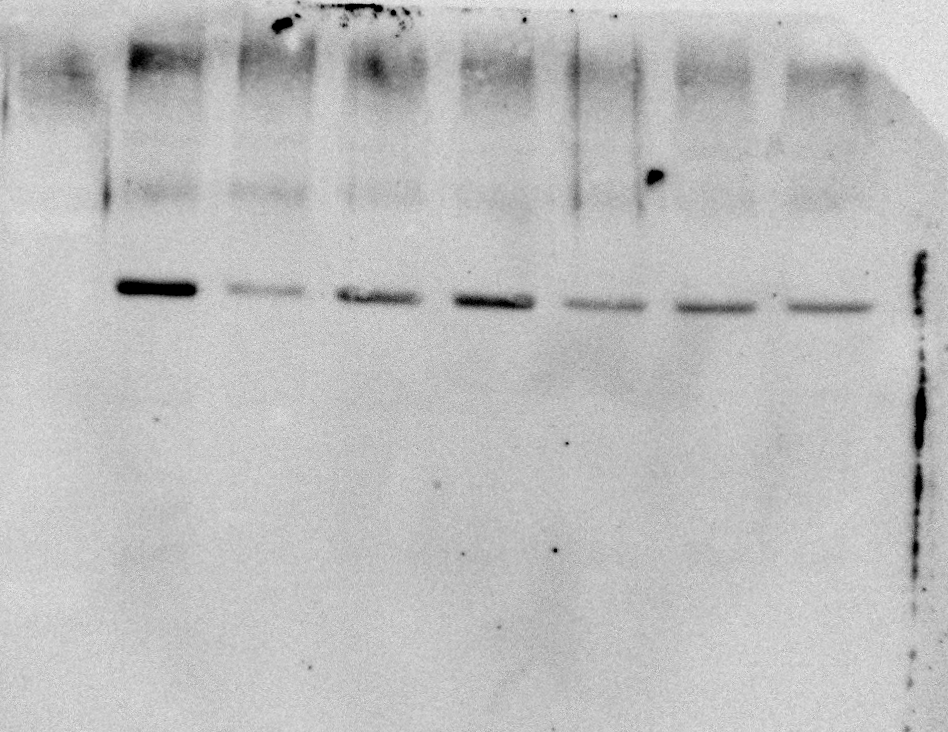 | 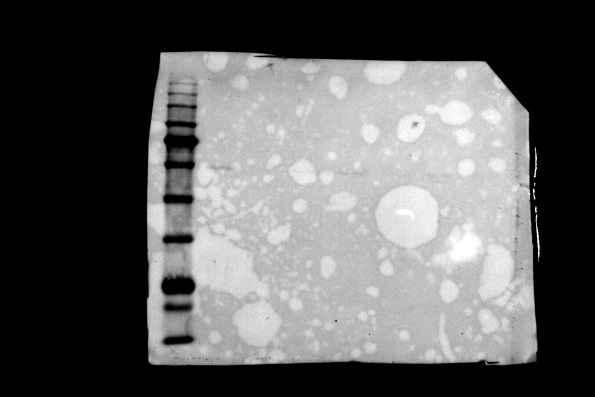  45kD  63kD |
|  | HFD.S | 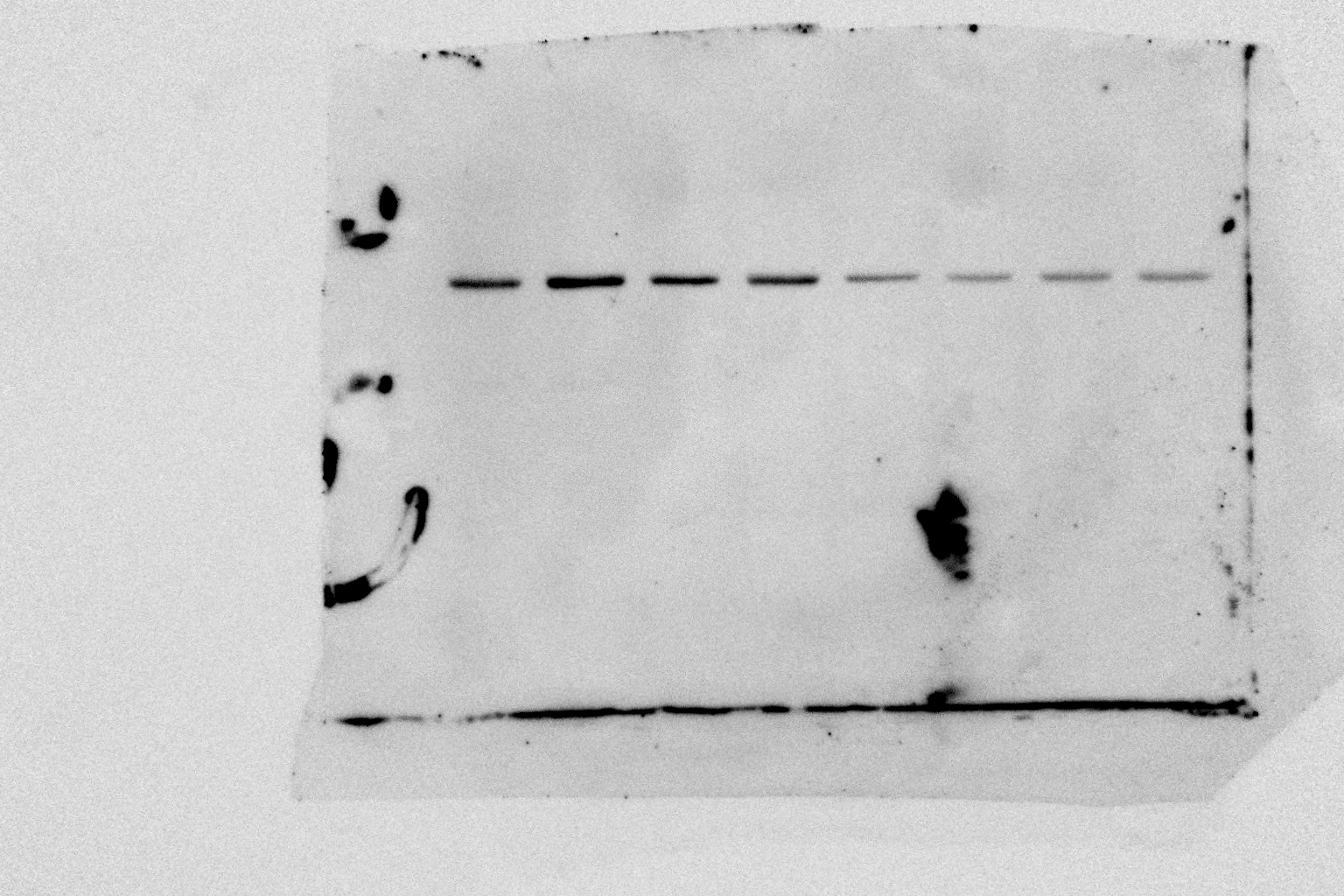 | 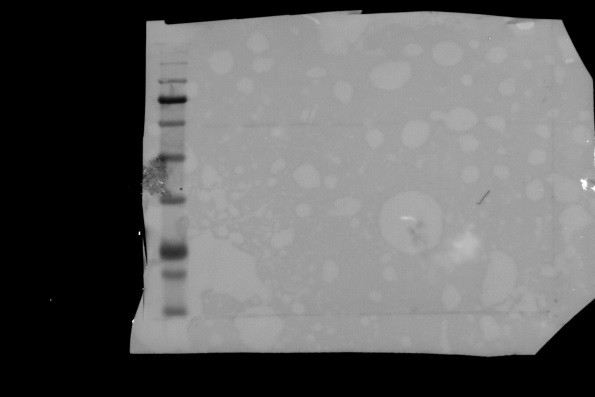  45kD  63kD |
| PKB | STD | 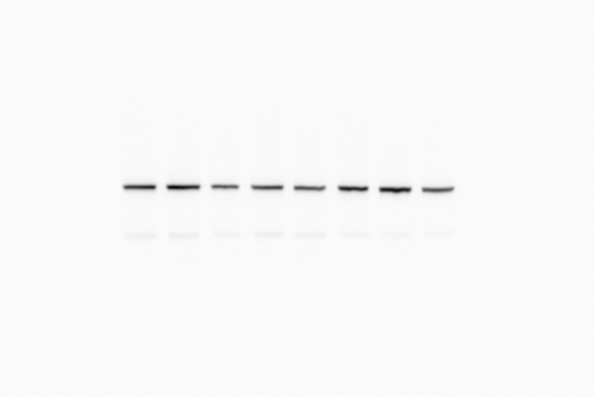  63kD  45kD | 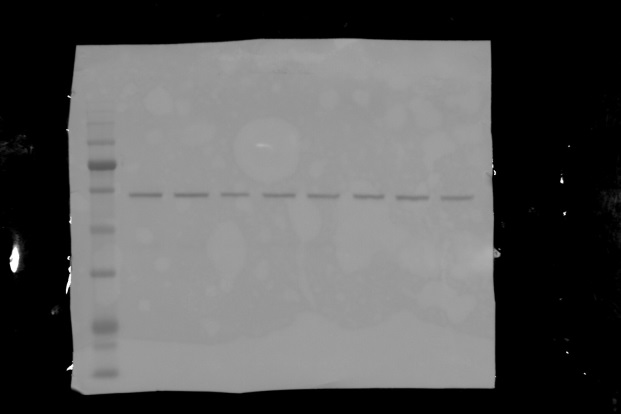 |
|  | HFD | 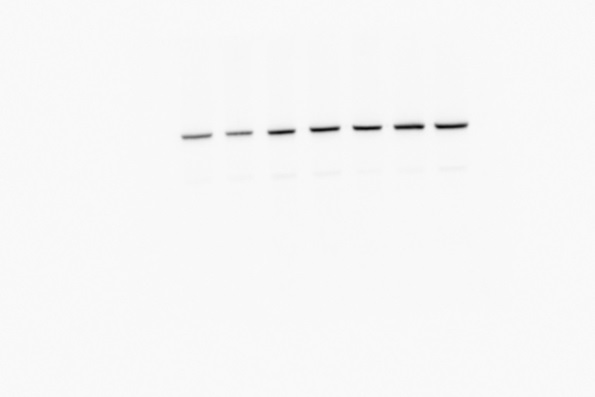 | 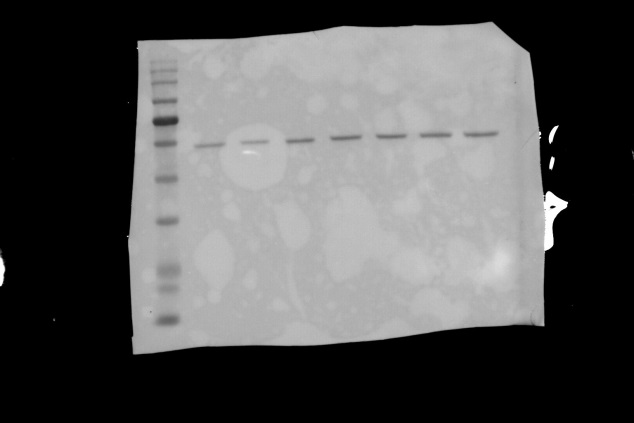  45kD  63kD |
|  | HFD.S | 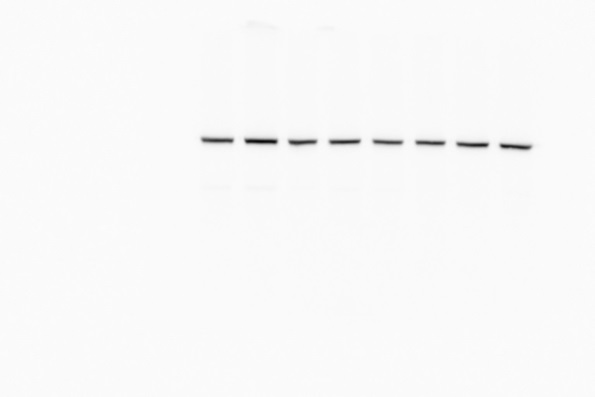 | 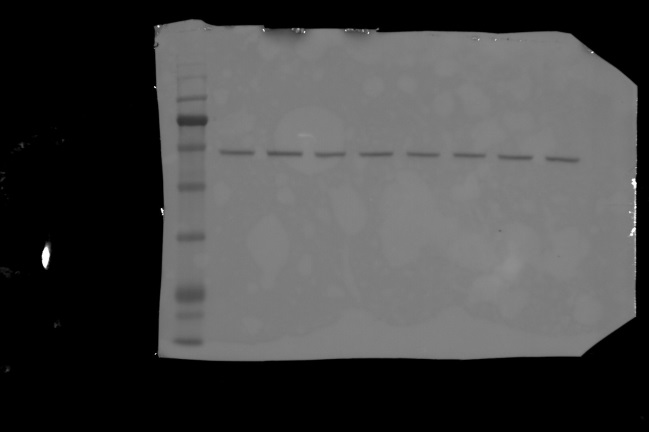  45kD  63kD |
| pGSK | STD | 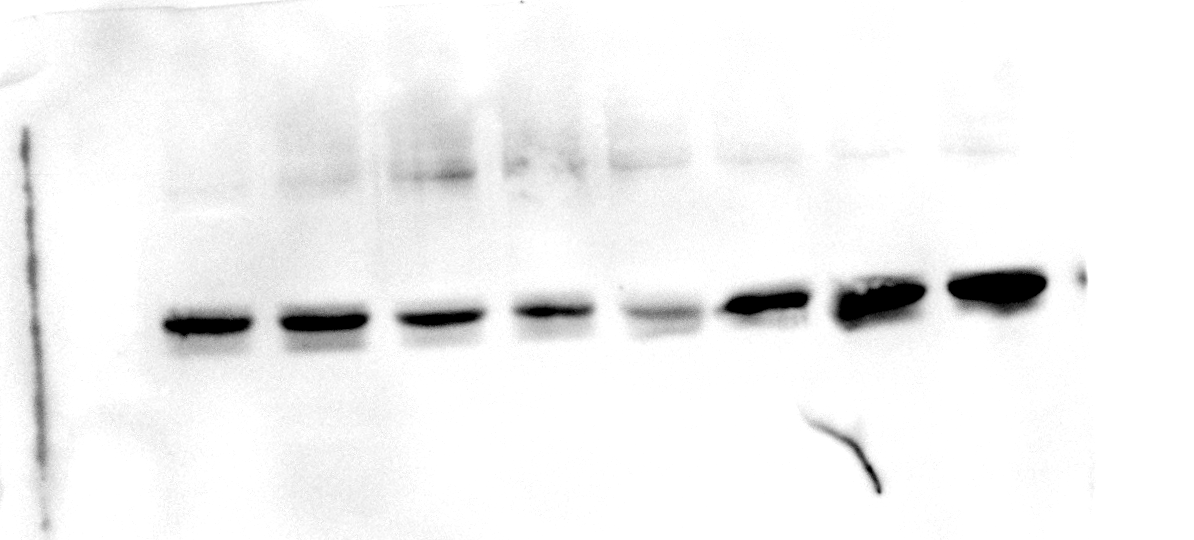 | 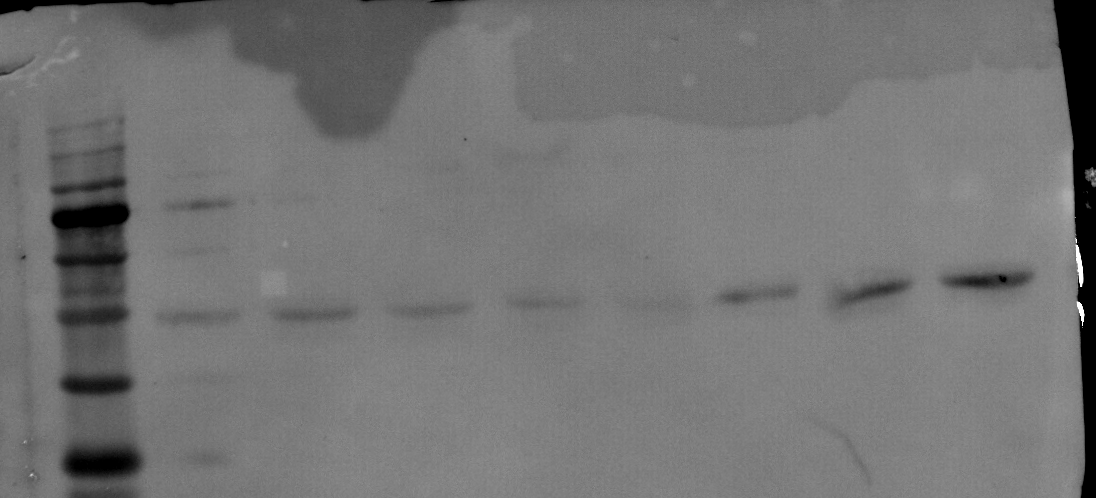  37kD  45kD |
|  | HFD | 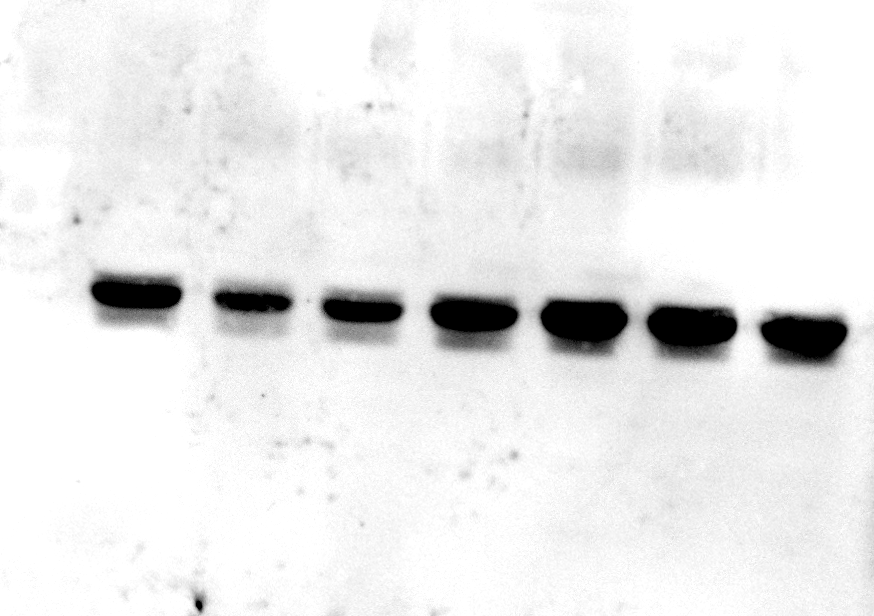 | 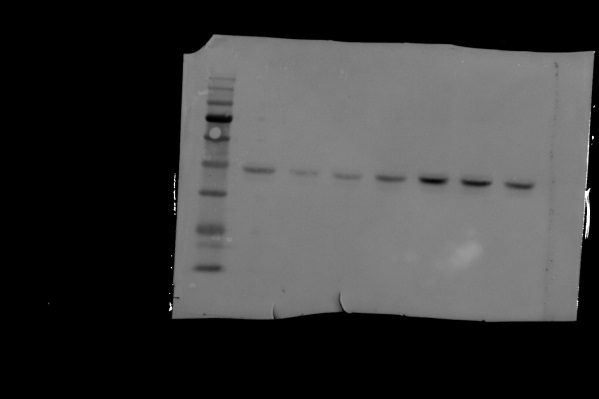  37kD  45kD |
|  | HFD.S | 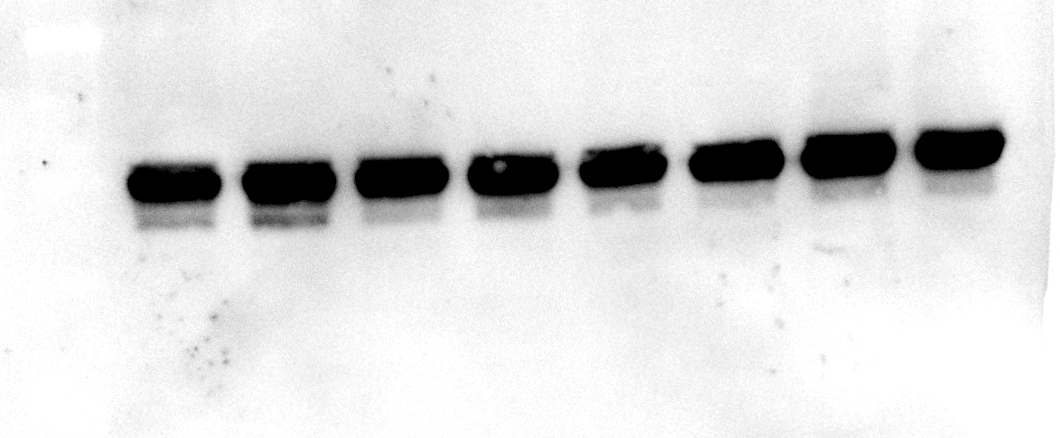  45kD  37kD | 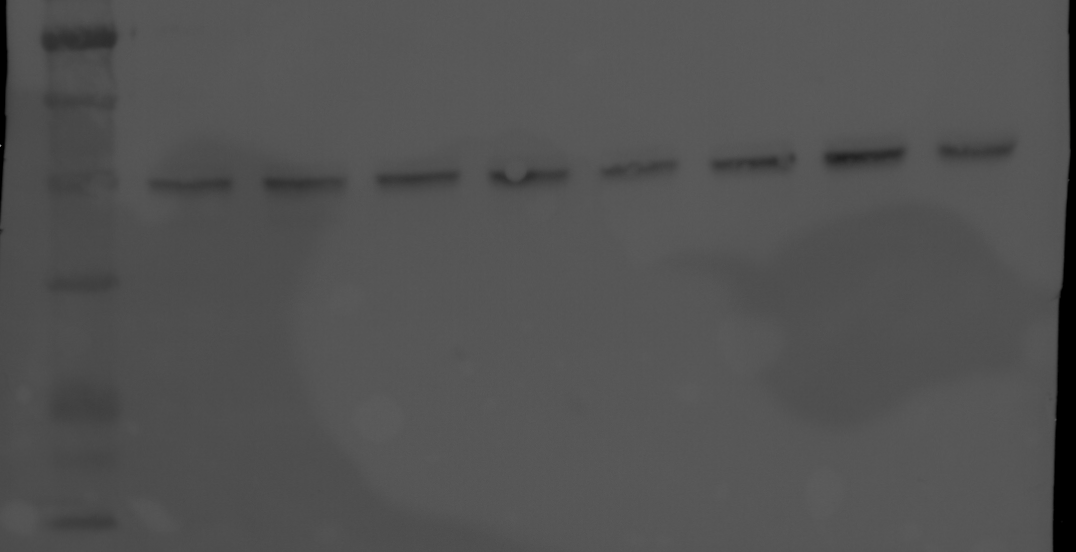 |
| GSK | STD | 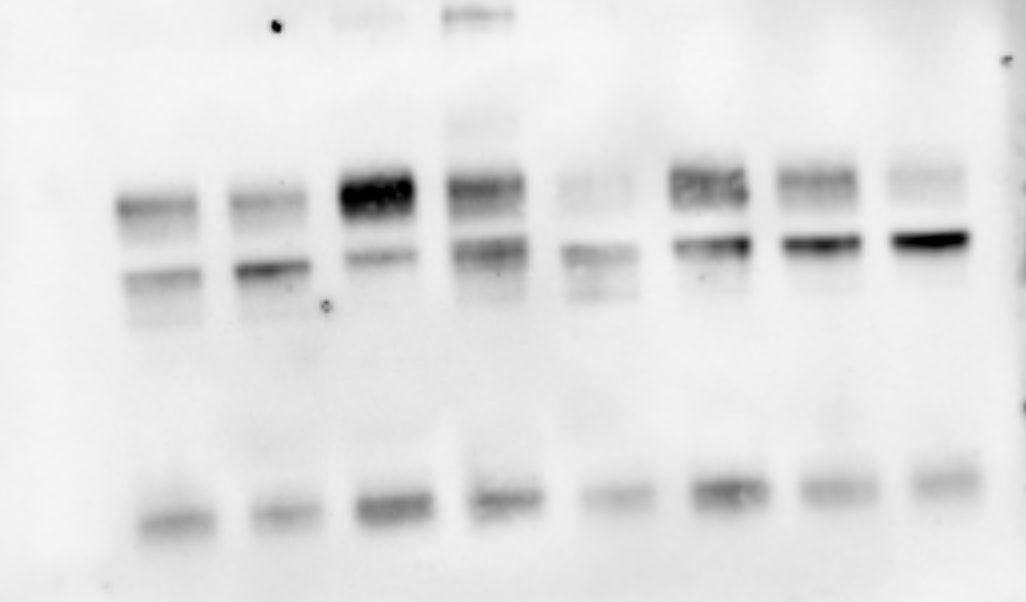 | 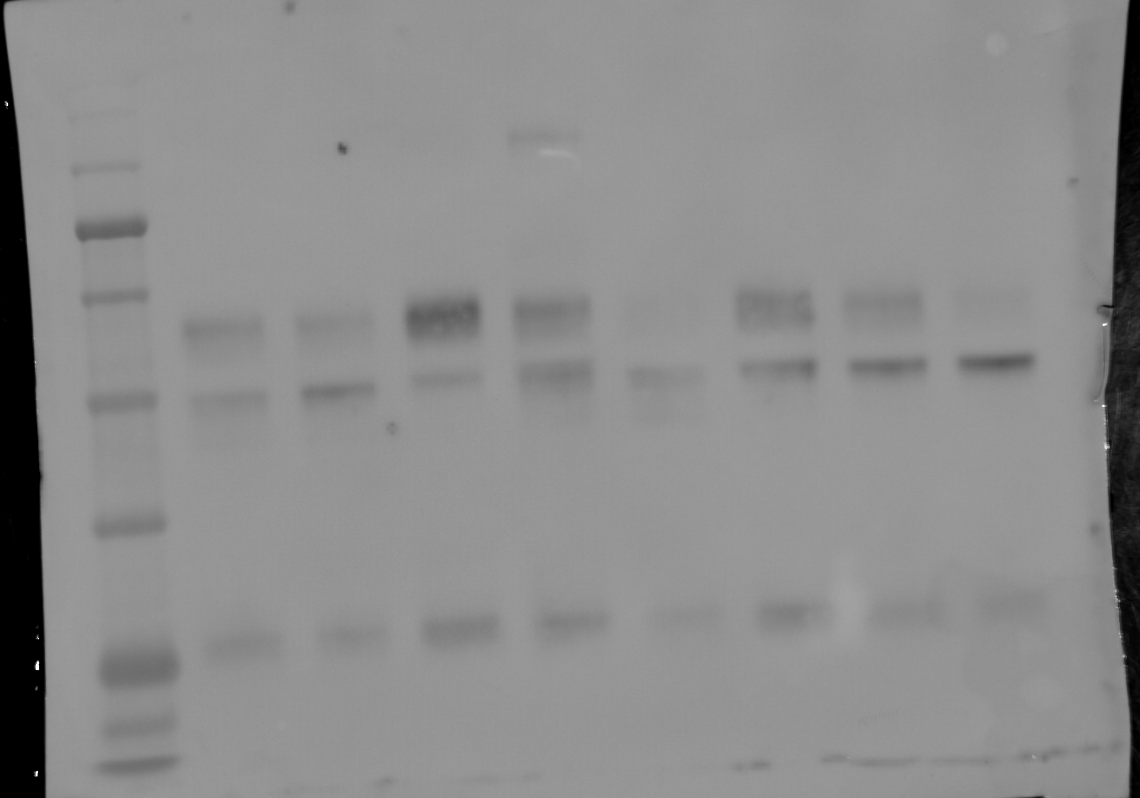  37kD  45kD |
|  | HFD | 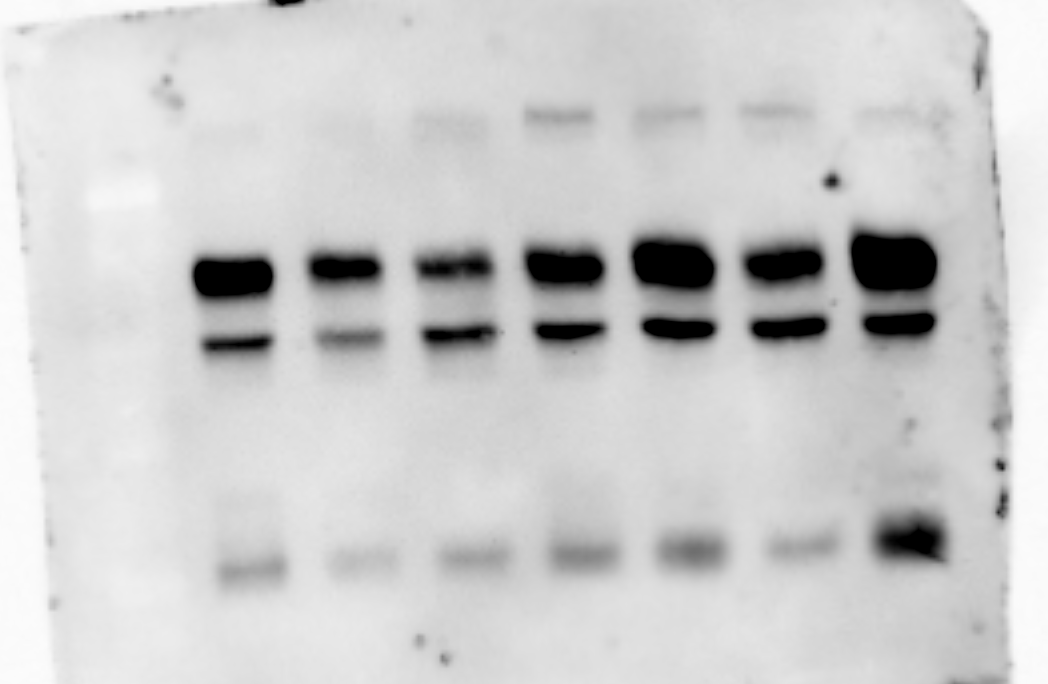 | 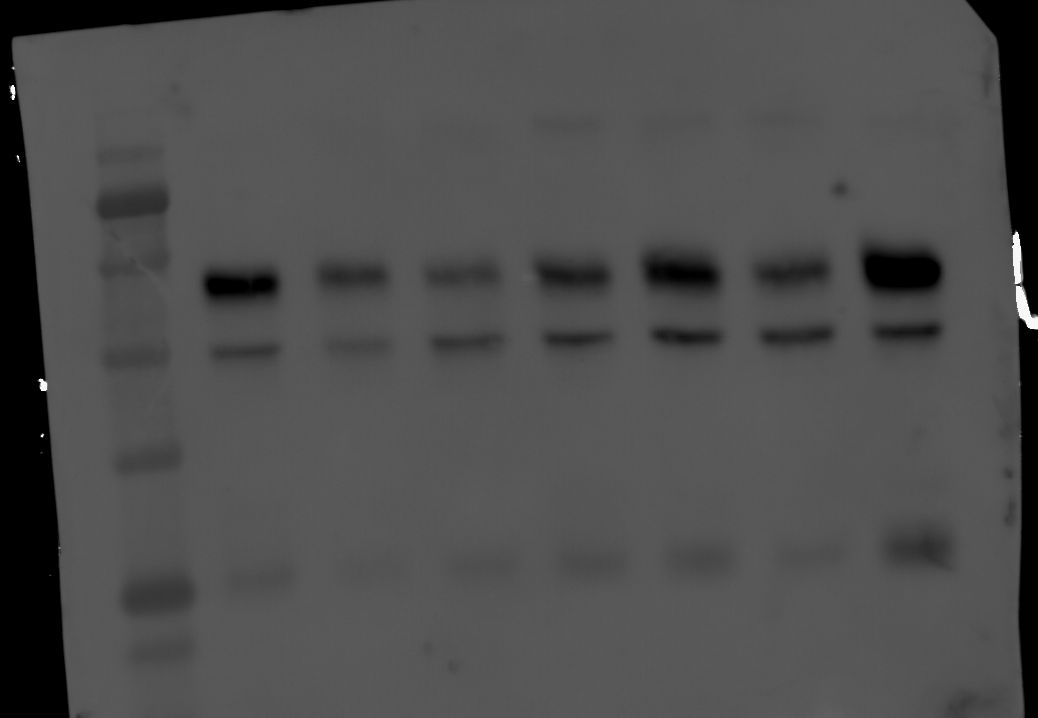  45kD  37kD |
|  | HFD.S | 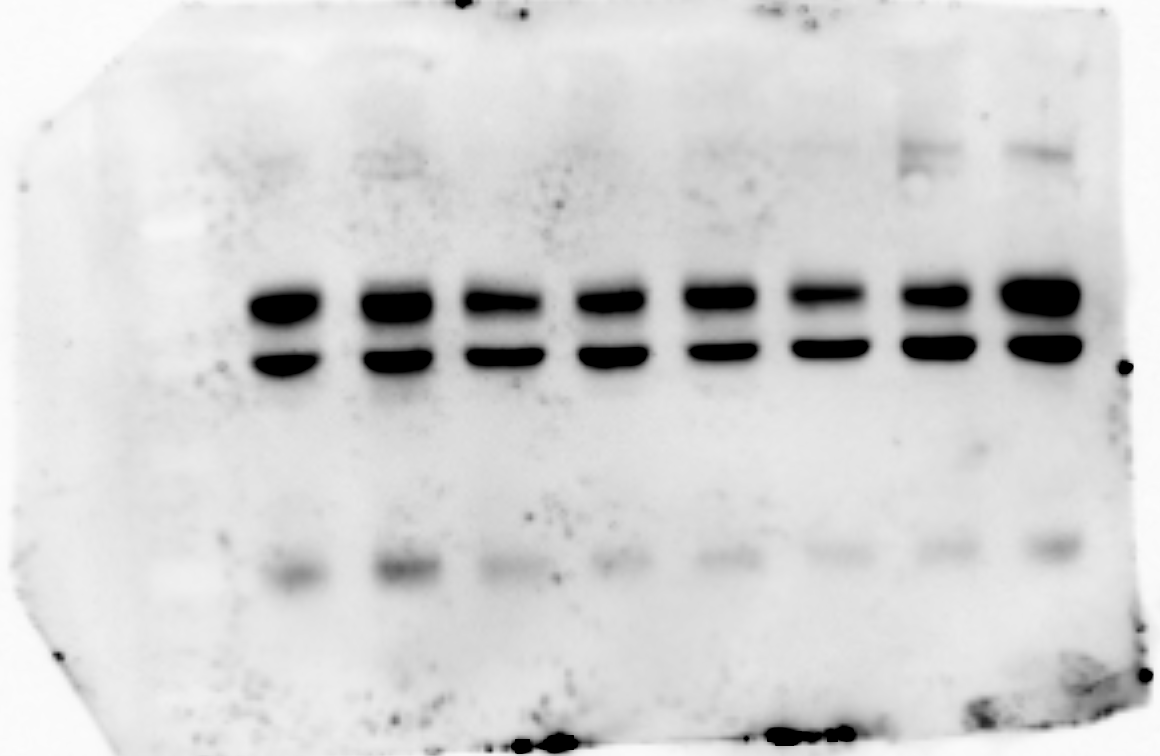  37kD  45kD | 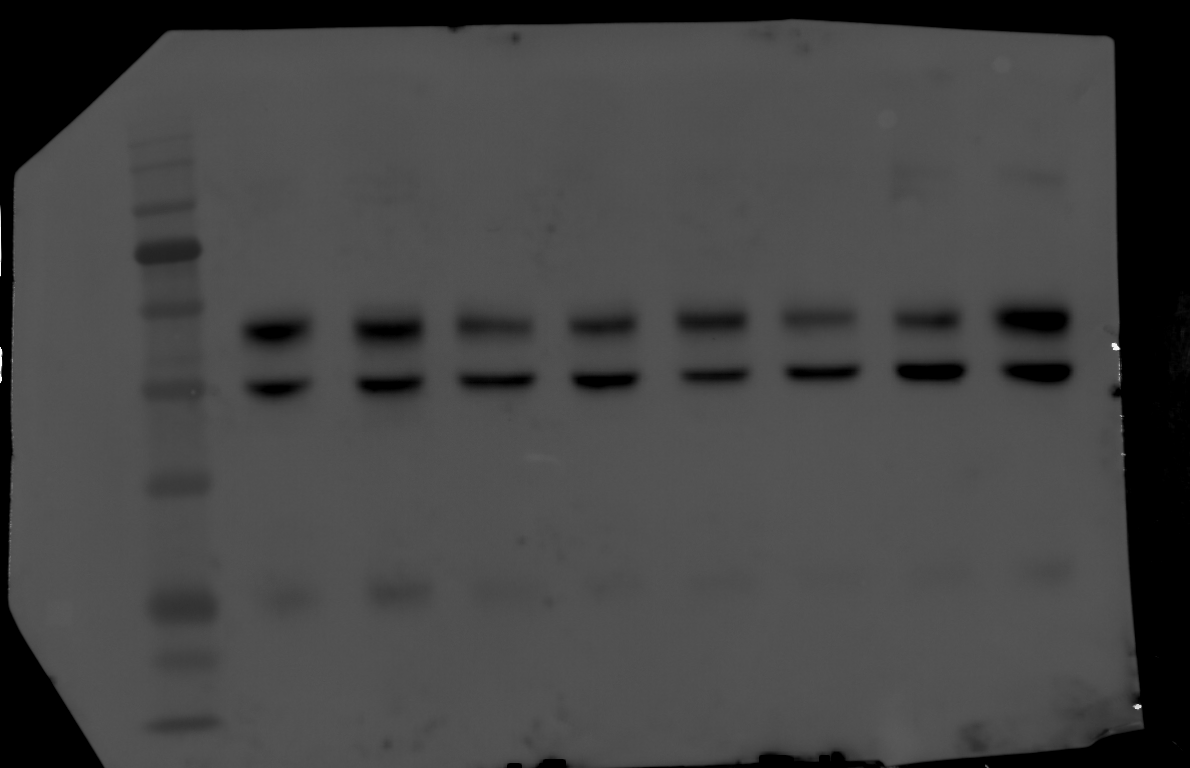 |
| pPRAS | STD | 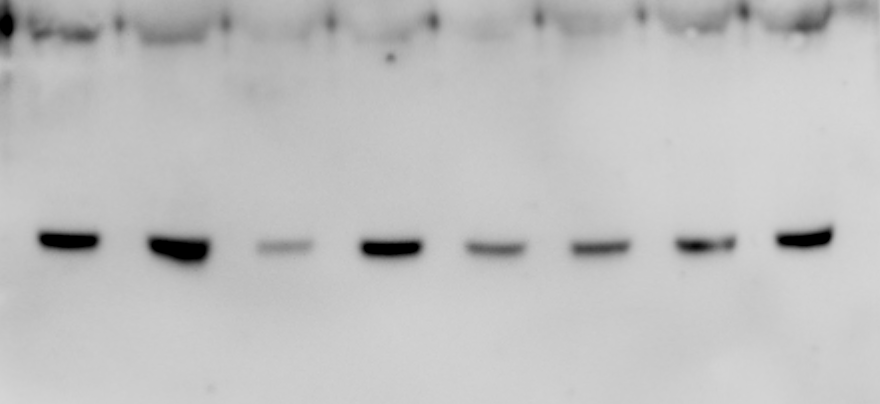 | 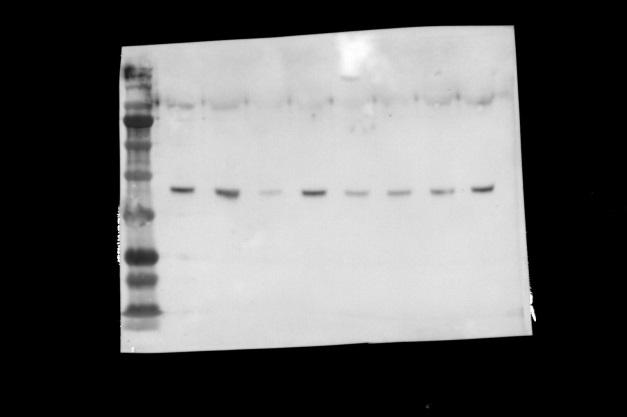  35kD  45kD |
|  | HFD | 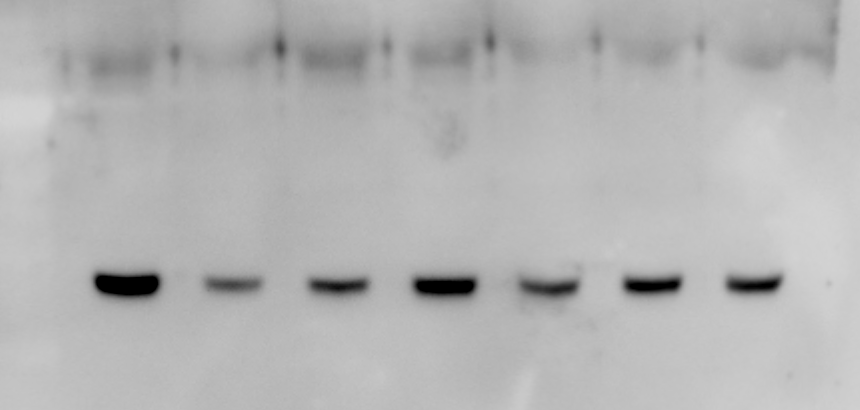 | 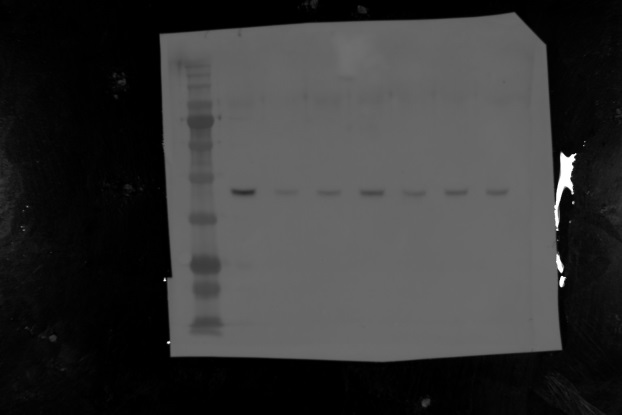  35kD  45kD |
|  | HFD.S | 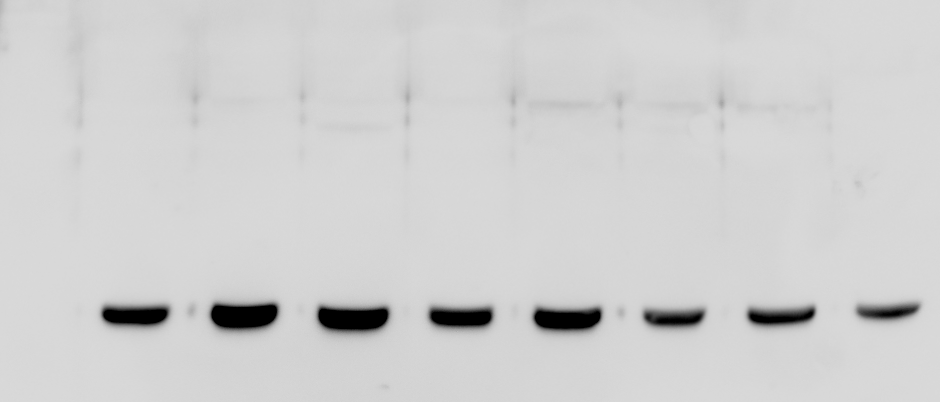 | 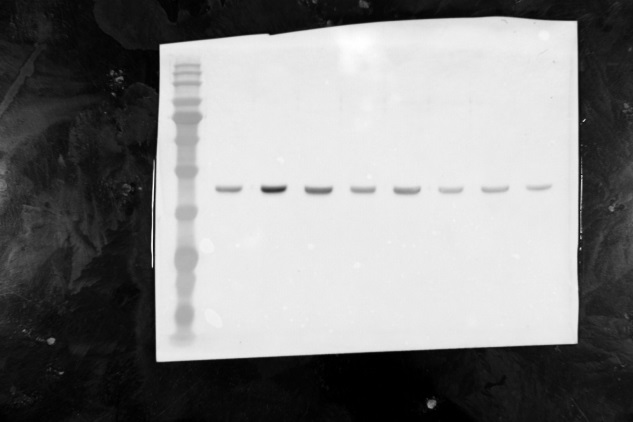  35kD  45kD |
| Actin | STD | 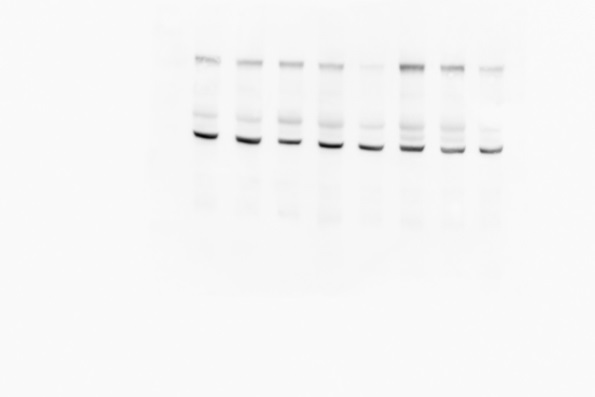 | 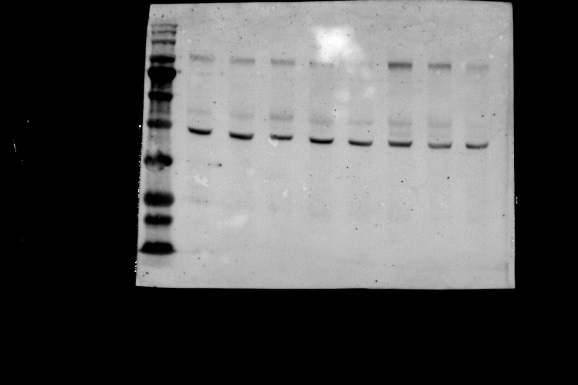  35kD  45kD |
|  | HFD | 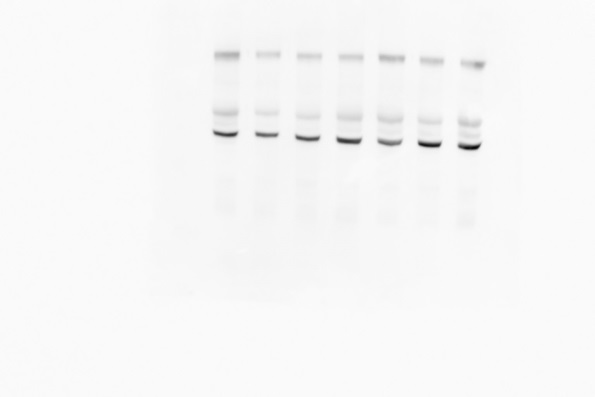 | 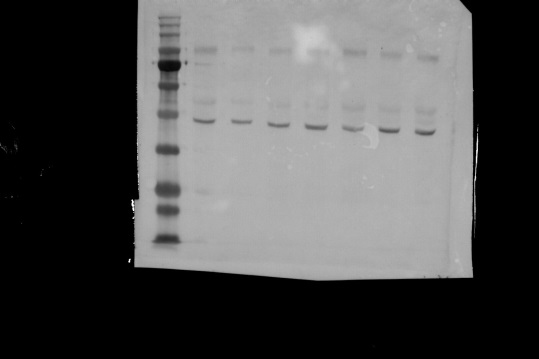  35kD  45kD |
|  | HFD.S | 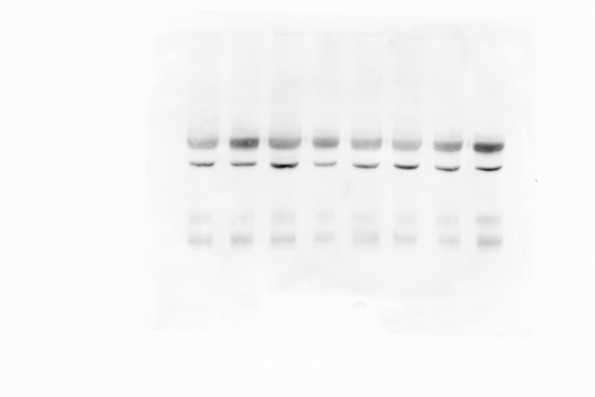 | 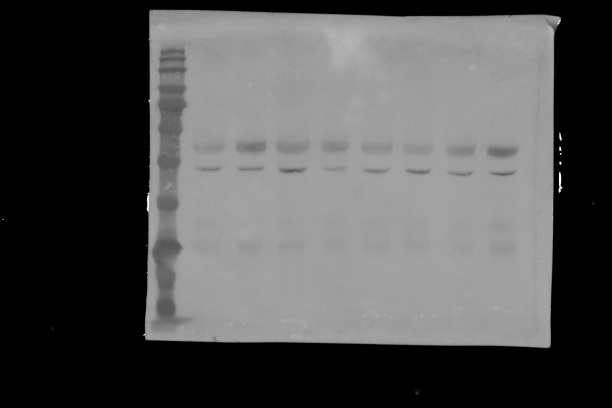  35kD  45kD |

Supplementary figure 2. Original blots presented in Figure 3. When a significant different in the molecular weight of protein of interest exists, some of the membranes were re-blotted with additional primary antibodies, thus the "non-specific bands" are the bands developed as a result of the previous primary antibody still exists, as is seen in the blot of pPKB.
