# Supplementary material for: Sarcopoterium spinosum extract improved insulin sensitivity in mice models of glucose intolerance and diabetes
Source: PLoS One. 2018 May 16;13(5):e0196736. doi: 10.1371/journal.pone.0196736 (PMC5955592; doi:10.1371/journal.pone.0196736)
Supplement: S3 Fig — A. Original blots presented in Fig 6A. B. Original blots presented in Fig 6B. In this set of blots, the order of loading was as followed: S. spinosum treated without insulin stimulation (3 mice), S. spinosum treated with insulin stimulation (3 mice), control mice without insulin stimulation (4 mice), control mice with insulin stimulation (4 mice). In order to present the results in more logical way, in Fig 5A and 5B the control bands were separated from S. spinosum bands and are presented at the left of the panel, without any manipulation of the results. (DOCX) [file pone.0196736.s003.docx]

Supplementary figure 3A.

|  | Chemiliuminesence | Merge (Chemiliuminesence+ bright field) |
| --- | --- | --- |
| pIR | 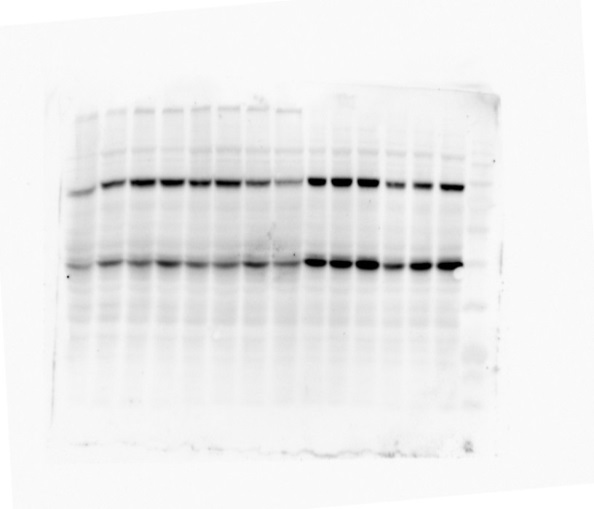 | 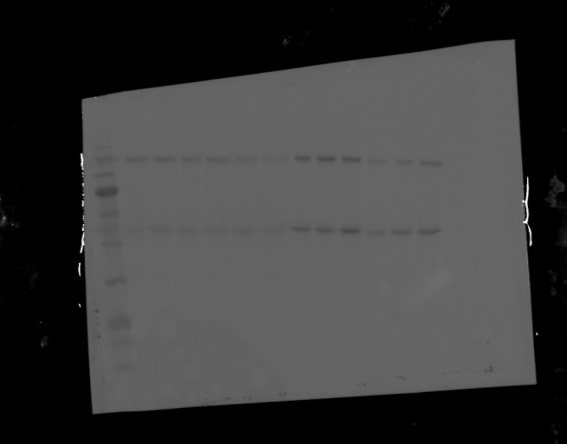  75kd  100kd |
| IR | 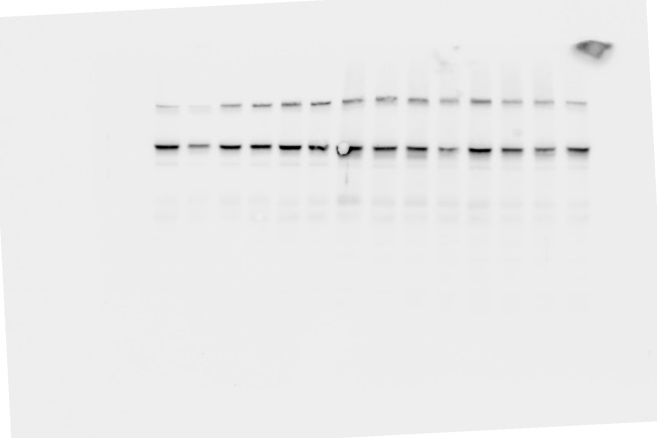 | 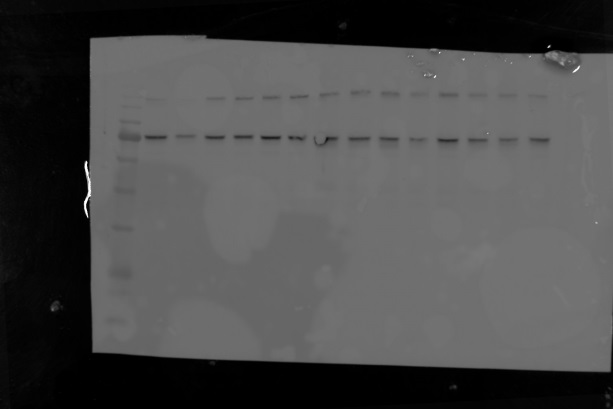  100kd  75kd |
| pPKB | 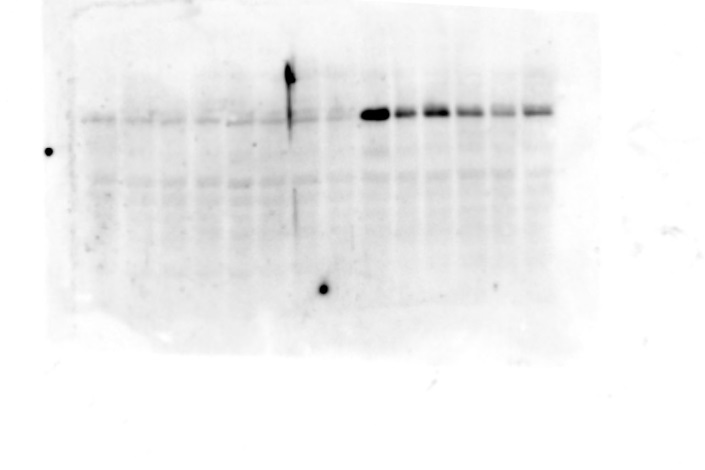 | 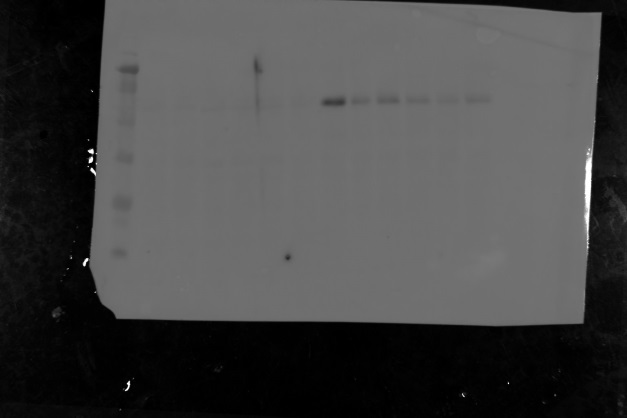  63kd  75kd |
| PKB | 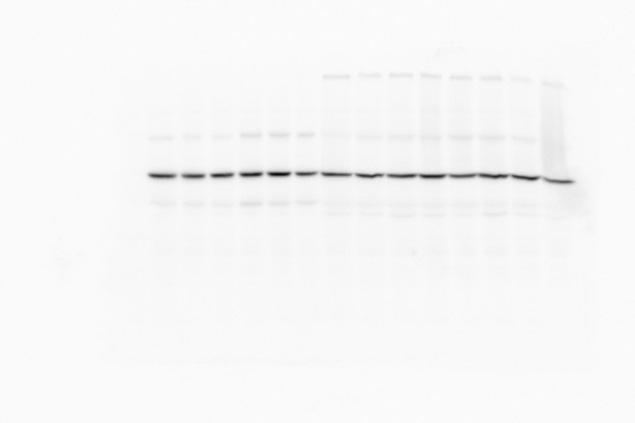 | 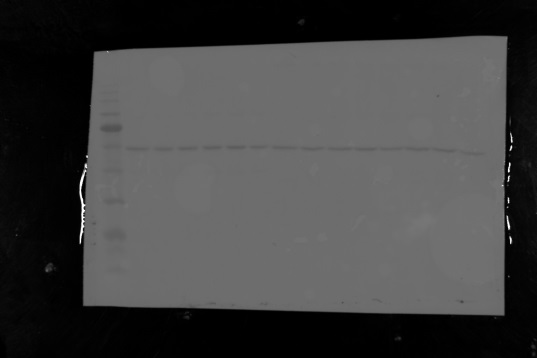  75kd  63kd |
| pGSK | 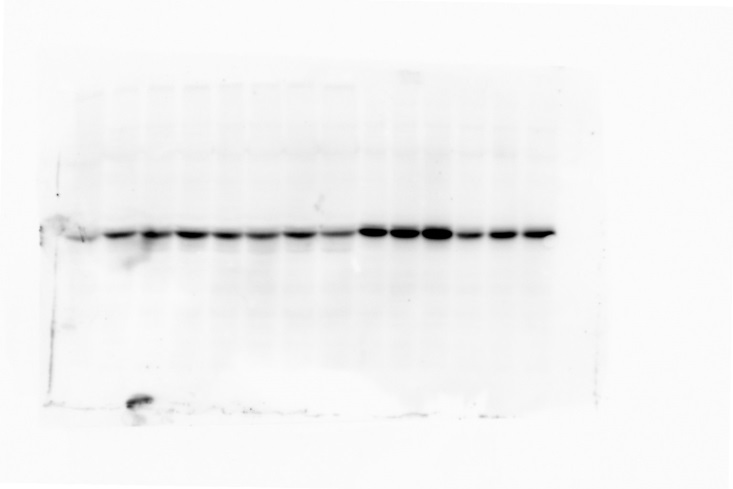  63kd | 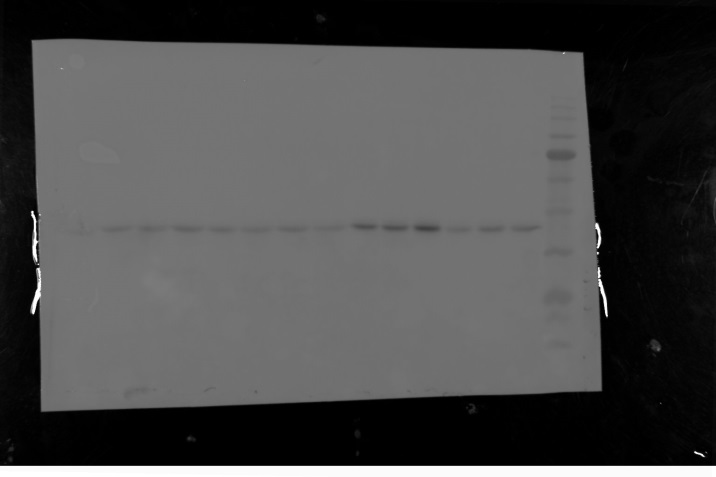  48kd |
| GSK | 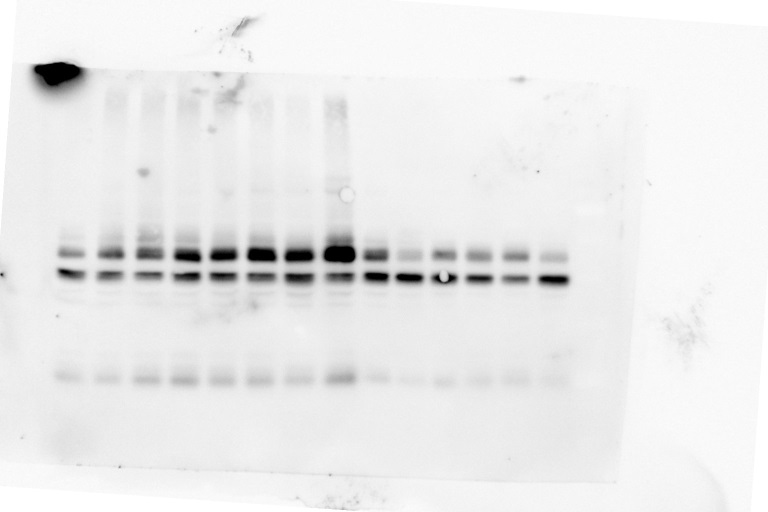 | 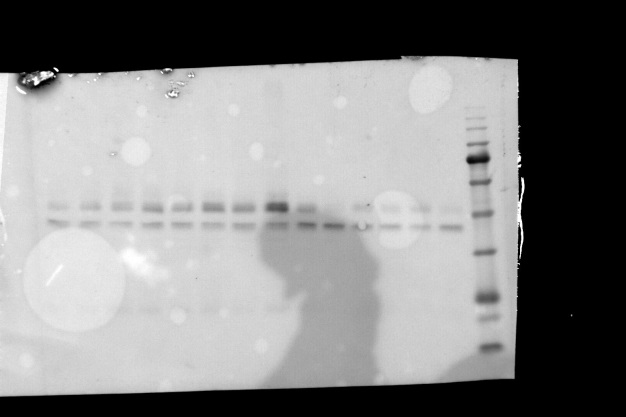  63kd  48kd |
| actin | 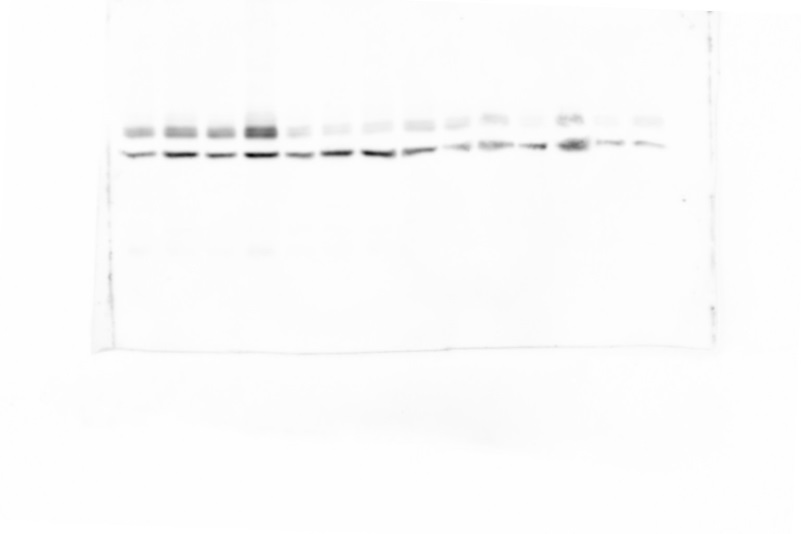 |   48kd  63kd |

Supplementary figure 3B

|  | Chemiliuminesence | Merge (Chemiliuminesence+ bright field) |
| --- | --- | --- |
| pIR | 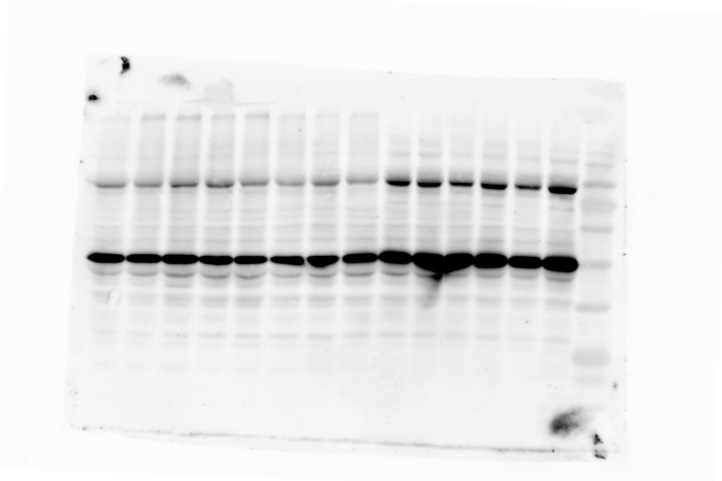  100kd  75kd | 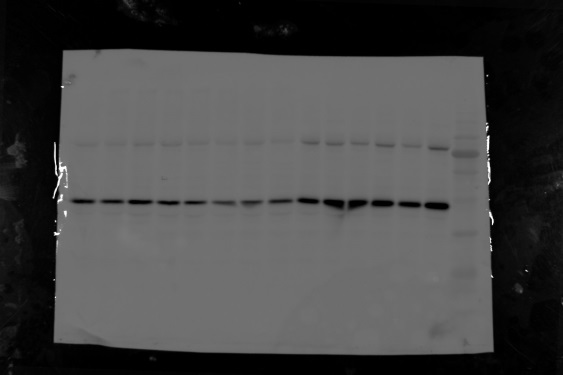 |
| IR | 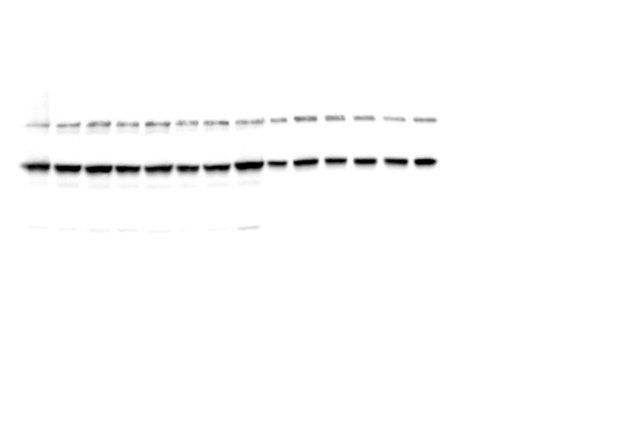 | 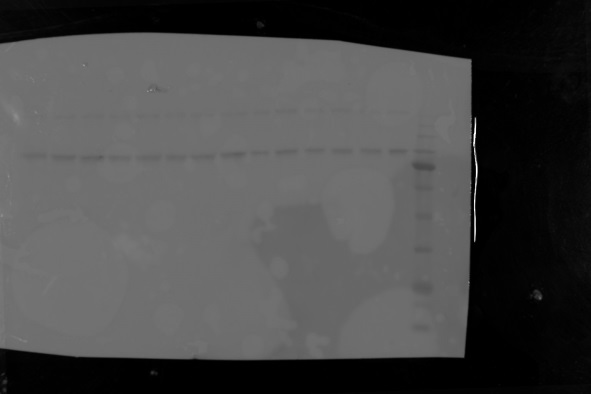  100kd  75kd |
| pPKB | 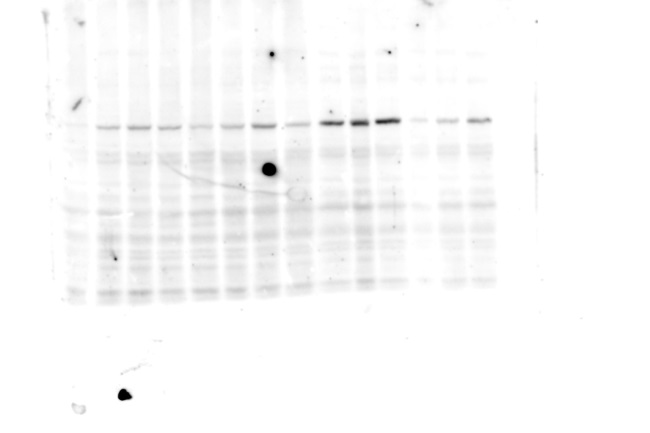  63kd  75kd | 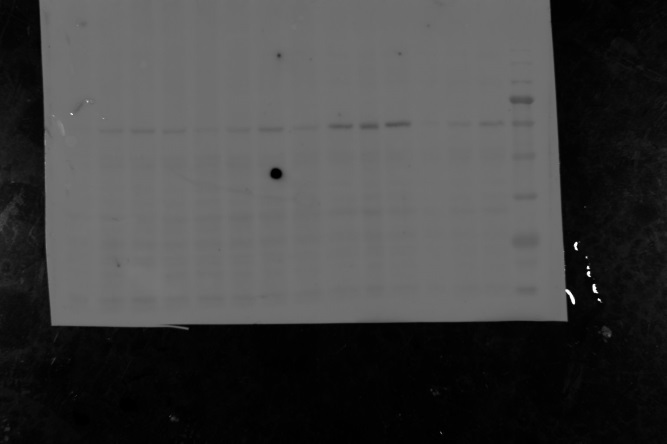 |
| PKB | 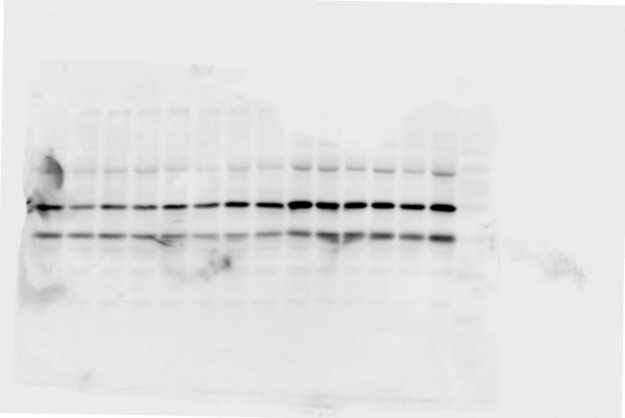  63kd  75kd | 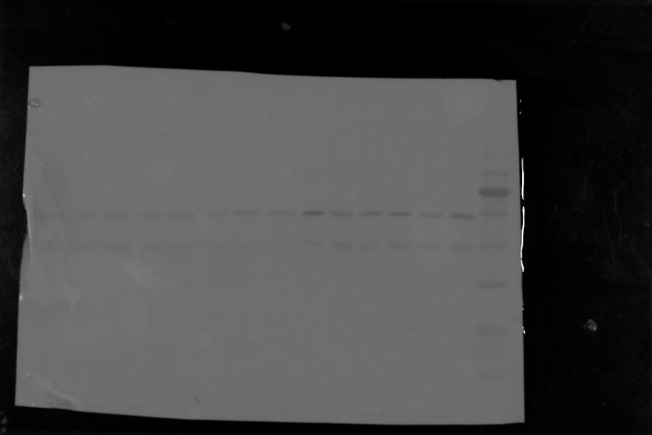 |
| pGSK | 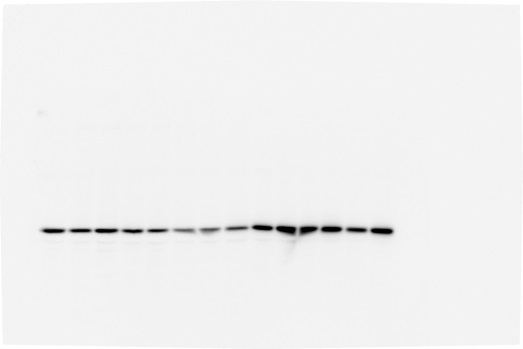  48kd  63kd | 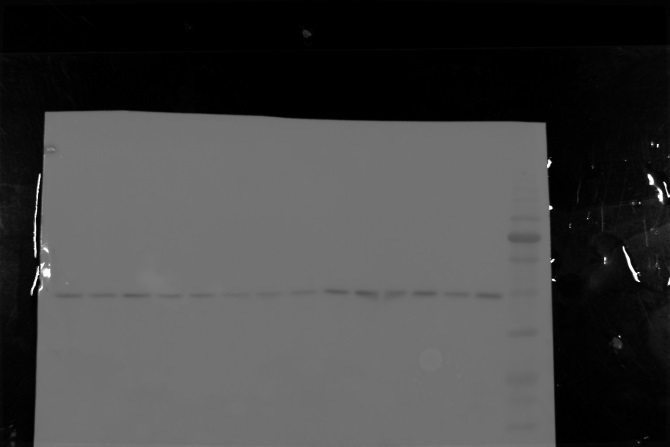 |
| GSK | 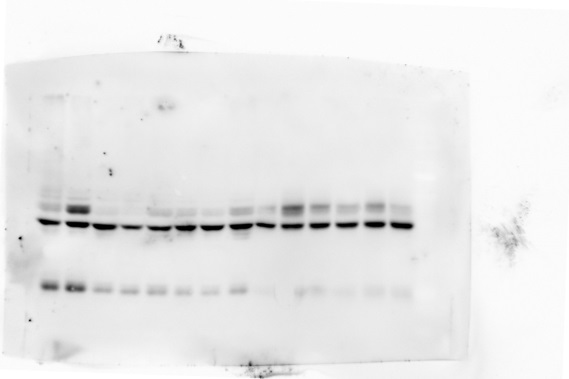  48kd  63kd | 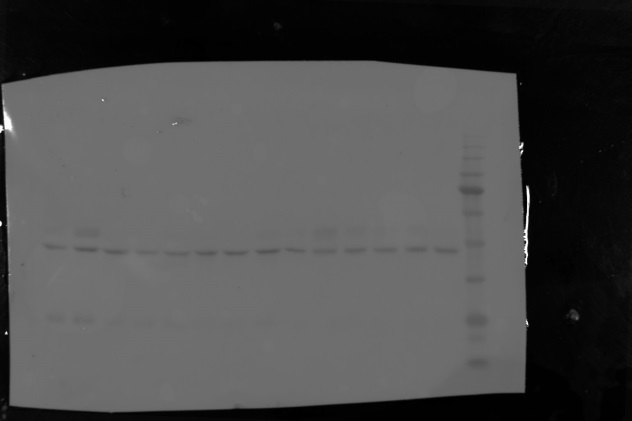 |
| actin | 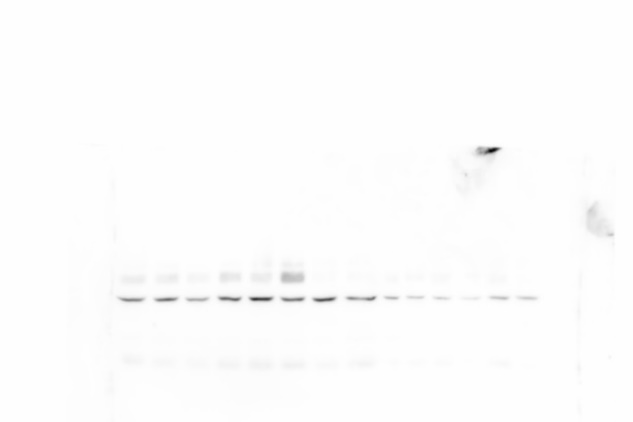 | 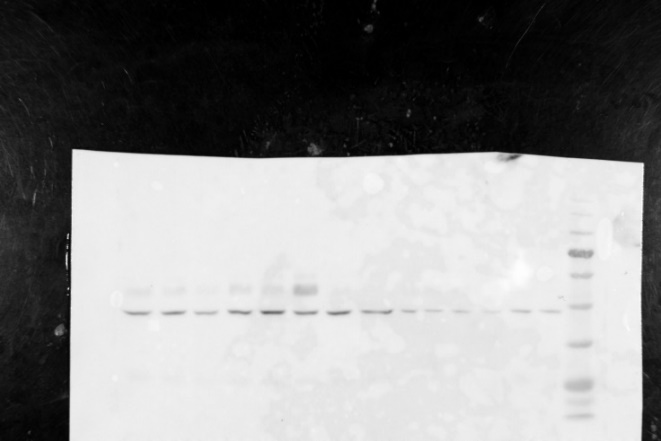  48kd  63kd |

**Supplementary figure 3**. Original blots presented in Figure 6. A. Original blots presented in Fig 6A. B. Original blots presented in Fig 6B. In this set of blots, the order of loading was as followed: *S. spinosum* treated without insulin stimulation (3 mice), *S. spinosum* treated with insulin stimulation (3 mice) and control mice without insulin stimulation (4 mice), control mice with insulin stimulation (4 mice). In order to present the results in more logical way, in Fig 5A and B the control bands were separated from *S. spinosum* bands and are presented at the left of the panel, without any manipulation of the results.
